# Favorable safety outcomes of a perioperative propranolol and etodolac regimen in cancer patients in four randomized controlled trials

**DOI:** 10.3389/fphar.2026.1823113

**Published:** 2026-04-20

**Authors:** Nahida Sakis, Liat Sorski, Eden Asraf, Itay Ricon-Becker, Lee Shaashua, Rita Haldar, Bar Bruno Shvalbo, Maytal Shabat-Simon, Mordechai Gutman, Aviram Nissan, Ido Nachmany, Ilan Kent, Ron Pery, Niv Pencovich, Noam Shussman, Nir Wasserberg, Avraham Reshef, Elchanan Quint, Yifat Yosef Lishtzinsky, Hanoch Kashtan, Moshe Shabtai, Baruch Brenner, Eran Sharon, Tanir Allweis, Nitzan Shahar, Anabel Eckerling, Elad Sandbank, Oded Zmora, Shamgar Ben-Eliyahu

**Affiliations:** 1 School of Psychological Sciences, Tel Aviv University, Tel Aviv, Israel; 2 Sagol School of Neuroscience, Tel Aviv University, Tel Aviv, Israel; 3 Department of Surgery and Transplantation, Sheba Medical Center, Ramat Gan, Israel; 4 Department of Surgical Oncology, Sheba Medical Center, Ramat Gan, Israel; 5 Department of General Surgery, Sheba Medical Center, Ramat Gan, Israel; 6 Department of General Surgery, Hadassah Medical Center and Faculty of Medicine, Hebrew University of Jerusalem, Jerusalem, Israel; 7 Department of Surgery, Rabin Medical Center, Beilinson Hospital, Petah Tikva, Israel; 8 Department of Surgery, Soroka University Medical Center, Beer Sheva, Israel; 9 Department of Colorectal Surgery, Assuta Ashdod Hospital, Ashdod, Israel; 10 Department of Oncology, Rabin Medical Center, Beilinson Hospital, Petah Tikva, Israel; 11 Department of Surgery, Kaplan Medical Center, Rehovot, Israel; 12 Department of Surgery, Shamir Medical Center, Beer Yaakov, Israel

**Keywords:** COX2 inhibitors, etodolac, metastatic disease, non-selective B-blockers, perioperative care, propranolol, randomized controlled trial, safety

## Abstract

**Background:**

The perioperative use of the β-adrenergic blocker, propranolol, and/or the semi-selective COX-2 inhibitor, etodolac, has improved biomarkers of cancer metastasis in several small randomized controlled trials (RCTs). In colorectal cancer (CRC) patients, the combined regimen showed potential to improve disease-free survival (DFS).

**Methods:**

We report data from our four RCTs including 148 patients with breast (n = 38), colorectal (n = 34 & n = 46), and pancreatic (n = 30) cancers. Treatment began 5 days pre-operatively, and continued for 5–30 postoperatively. Propranolol (slow-release) was initiated at 20 mg twice daily (b.i.d), increased to 80 mg b.i.d on surgery day, and gradually decreased thereafter to 20 mg b.i.d. Etodolac was provided at 400 mg b.i.d. The primary endpoints were perioperative safety outcomes, including adverse events (AEs) up to 30-day postoperatively, and 16 blood indices. Secondary outcomes were long-term oncological outcomes of 8-year DFS and overall survival (OS).

**Results:**

Bradycardia occurred in 12% of treated vs. 3% of placebo patients (p = 0.057), and was easily resolved by temporary withholding β-blockade. Weakness, nausea, pain, infection, bleeding, leakage, tissue-healing, or death were not significantly affected. Drug treatment promoted eosinophils in pancreatic cancer patients (p = 0.015), increased potassium (p = 0.013), and decreased albumin (p = 0.016) in CRC patients; however, these effects did not remain significant after false discovery rate (FDR) correction. In CRC, treatment improved 8-year DFS (2/15 vs. 9/18, p = 0.034), although this exploratory analysis was underpowered.

**Conclusion:**

These findings suggest the safety of this inexpensive and easy-to-implement perioperative propranolol and etodolac regimen in patients participating in these RCTs.

**Clinical Trial Registration:**

https://clinicaltrials.gov/study/NCT00502684, https://clinicaltrials.gov/study/NCT00888797, https://clinicaltrials.gov/study/NCT03919461, https://clinicaltrials.gov/study/NCT03838029, identifier NCT00502684, NCT00888797, NCT03919461, NCT03838029.

## Introduction

In 2022, ∼20 million people were newly diagnosed with cancer, and cancer mortality mounted to ∼9.7 million worldwide (Bray et al., 2024). Most solid cancer patients undergo curative surgery for the excision of a primary tumor (Miller et al., 2022), but a significant percentage of these patients will develop metastatic disease, which is the major cause of cancer-related death (Dillekas et al., 2019). Unfortunately, surgical excision of a primary tumor, although essential and life-saving, may also accelerate metastatic processes, including the development of pre-existing micrometastases, and spreading and seeding of tumor cells (Horowitz et al., 2015; Hiller et al., 2018; Ricon et al., 2019). Importantly, the immediate perioperative period (IPP), days before and after surgery, including surgery itself, induces significant pro-, as well as anti-metastatic processes (Ben-Eliyahu, 2020). The balance between these opposing processes often determines whether minimal residual disease (MRD) will be eliminated, remain dormant, or progress and become more resistant, sustainable, self-propagating, and eventually erupt in the form of overt metastases (Ben-Eliyahu, 2020; Shaashua et al., 2020). Therefore, the perioperative period is now recognized as having a marked impact on long-term cancer outcomes, despite its relatively short duration (Horowitz et al., 2015; Ben-Eliyahu, 2020; Eckerling et al., 2021; Ergun, 2024).

Several factors during the perioperative period were shown to support the survival of remaining cancer foci and/or accelerate their progression, by (i) directly affecting malignant tissue and its microenvironment, and (ii) by suppressing anti-metastatic cell-mediated immunity (CMI) (Horowitz et al., 2015; Shakhar and Ben-Eliyahu, 2003; Neeman et al., 2012; Neeman and Ben-Eliyahu, 2013; Cole et al., 2015). Based on existing literature, including our recent translational and clinical trials in breast cancer (BC) and colorectal cancer (CRC) patients (Haldar et al., 2020; Haldar et al., 2018; Shaashua et al., 2017), catecholamines (CAs) and prostaglandins (PGs) have emerged as key mediators of the deleterious effects of stress and surgery during the IPP (Horowitz et al., 2015; Ricon et al., 2019; Rosenne et al., 2014). Recently, in several small randomized clinical trials (RCTs), we and others have used the β-adrenergic blocker, propranolol, and/or the COX-2 synthesis inhibitor, etodolac, to perioperatively inhibit CAs and PGs signaling. The drugs consistently showed positive impact on prognostic biomarkers of cancer progression in excised tumors, including reduced epithelial-to-mesenchymal transition (EMT), and reduced GATA and STAT transcriptional activity (Haldar et al., 2020; Haldar et al., 2018; Shaashua et al., 2017; Knight et al., 2018; de la Torre et al., 2015). Moreover, in CRC patients, the propranolol-etodolac regimen also significantly improved 5-year disease-free survival (DFS) (Ricon-Becker et al., 2023), although this small RCT was not powered to assess this outcome. Thus, limiting CAs and PGs signaling during the IPP should be studied on a larger scale to test its promising potential to improve long-term cancer outcomes (Horowitz et al., 2015; Hiller et al., 2018; Eckerling et al., 2021; Neeman and Ben-Eliyahu, 2013).

It is important to recognize that in the perioperative context, both CAs and PGs are simultaneously released. Many tumor types secrete PGs, which suppress CMI(4, 12). Additionally, levels of stress-inflammatory related factors are profoundly increased even before surgery due to anticipatory anxiety and stress (Shaashua et al., 2017), and their secretion is further increased due to surgery-related tissue damage, anesthesia, hypothermia, blood loss, nociception, and fatigue (Horowitz et al., 2015; Neeman et al., 2012). Since CAs and PGs are simultaneously elevated perioperatively, and as each ligand alone is sufficient to promote pro-metastatic processes through common intracellular pathways (e.g., elevated cAMP) (Horowitz et al., 2015; Neeman et al., 2012; Neeman and Ben-Eliyahu, 2013), we have hypothesized that their simultaneous blockade may be necessary to effectively prevent the deleterious pro-metastatic effects of surgical stress (Horowitz et al., 2015; Benish et al., 2008). Indeed, our preclinical studies have indicated an additive effect of the drugs, while in several studies only the simultaneous use of both drugs was effective (Benish et al., 2008; Melamed et al., 2005; Sorski et al., 2016; Glasner et al., 2010). For example, in melanoma tumor-bearing mice, only the perioperative combination of propranolol and etodolac improved long-term survival rates, while each drug alone was ineffective (Glasner et al., 2010). Therefore, to maximize the potential success of such a clinical intervention, our previous and ongoing RCTs employ the combination of the two drugs.

This study aims to evaluate the safety of perioperative propranolol-etodolac interventions to support large-scale clinical trials in heterogenous populations worldwide. Although these drugs have been used clinically for decades for various medical indications, yielding a good safety profile, there are risks and concerns associated with using these drugs in various medical settings, including in the oncological perioperative context. For etodolac, these include elevated risk for bleeding, disruption of tissue healing, anastomotic leakage, cerebrovascular accidents (CVA). For propranolol, these include bradycardia, and cardiovascular complications (Bakker et al., 2016; Group et al., 2008). To the best of our knowledge, there are no known clinically significant drug-drug interactions between propranolol and etodolac. Propranolol is used to treat tachycardia and is thus expected to reduce heart rate (HR) and blood pressure (BP); hence, bradycardia is expected in some patients during treatment. The safety results of the combined drug treatment presented herein are intended to inform future risk-benefit assessments. Of note, we have previously conducted animal studies testing the safety of separate and combined use of propranolol and etodolac in the perioperative context. These studies indicated no deleterious impacts on anastomotic integrity and tissue healing, and the general wellbeing of the animals (Benjamin et al., 2010; Hazut et al., 2011). We have now completed the treatment of 148 operated cancer patients with propranolol-etodolac (n = 76), or with placebo (n = 72) regimen, in four RCTs in patients with BC (RCT completed), CRC (one completed RCT and one ongoing RCT), or pancreatic cancer (PC; ongoing RCT). Safety was assessed based on (i) perioperative adverse events (AEs), (ii) blood biomarkers potentially related to perioperative complications (in the CRC and PC cohorts), and (iii) long-term cancer outcomes- DFS and overall survival (OS) in the CRC cohort. We herein report safety analyses for these 148 patients, both separately for each RCT/cancer type and as a combined dataset.

## Materials and methods

### Patient cohorts

Baseline characteristics, including age, sex, and relevant clinical history, were collected for all participants to ensure comparability between treatment arms (Table 1). Full demographics and CONSORT for the completed breast cancer and colorectal cancer studies have been published (Haldar et al., 2020; Shaashua et al., 2017). The pooled CONSORT of the four RCTs is provided in the supplementary (Supplementary Figure S3).

**TABLE 1:** Summary of patient cohorts and study parameters across our completed and ongoing RCTs examining perioperative treatment with propranolol and etodolac.

Cancer type	Stage	Total patient #	Recruit years	Sex	Age	Age: Median	# of medical centers (Israel)	NCT #
Breast	I-III, M0^a^	38	2013-2015	38 females	33–70	55	3; Kaplan, Sheba, Rabin	NCT00502684
Colorectal	I-III, M0^a^	34	2014-2016	17 females, 17 males	30-77	59	1; Sheba	NCT00888797
Colorectal	I-III, M0^a^	46	2019-present (on-going)	22 females, 24 males	33-81	57	6; Shamir, Rabin, Sheba, Soroka, Hadassah, Assuta	NCT03919461
Pancreatic	I-II, M0^a^	30	2019-present (on-going)	11 females, 19 males	43-80	64	2; Shamir, Sheba	NCT03838029

^a^ M0 in the TNM, staging system for cancer indicates that there is no evidence of distant metastasis.# indicates number (number of patients or medical centers).

### Patient screening and enrollment

Patients referred to surgery for the excision of BC, CRC, or PC with curative intent at the participating centers were considered for recruitment. The preoperative screening visit included a checklist of medical inclusion and exclusion criteria, and a detailed explanation of the study aims, procedures, potential benefits, and risks. Eligible patients who signed an informed consent form were recruited.

### Inclusion and exclusion criteria

Inclusion criteria included: (i) patients diagnosed with a single primary tumor and no evidence of metastatic disease, (ii) ASA physical status score of 1–3. Exclusion criteria included: (i) any contraindication for the drugs, including active asthma, peptic disease, and cardiovascular disease, (ii) patients with bradycardia (HR lower than 50 bpm) (iii) chronic use of any β-blocker or COX inhibitor, (iv) chronic autoimmune disease, and (v) known metastatic disease. All study protocols were approved by Institutional Review Boards (IRB) at each study site, as well as by the Ethics committee of Tel Aviv University, and written informed consent was obtained from patients before performing any study-related procedures.

### Treatment protocols and compliance monitoring

All four RCTs are designed as two-arm, double-blind, placebo-controlled trials. The total treatment period with the drug or placebo was 11 days for BC patients, 20 days for CRC patients, and 35 days for PC patients, starting 5 days before the surgery for tumor resection and including the day of surgery. Oral etodolac (Etopan®, Super-Pharm Professional Manufacturing, Israel) was administered at a dose of 400 mg twice daily (b.i.d) throughout the entire treatment period. Oral slow-release propranolol (Deralin® SR, Super-Pharm Professional Manufacturing, Israel) was given at 20 mg b.i.d for the 5 days before surgery, and 80 mg b.i.d on the day of surgery. Postoperative dosing varied: BC patients received 20 mg b.i.d. for an additional 5 days; CRC patients received 40 mg b.i.d. for the first 7 postoperative days, followed by 20 mg b.i.d. for the next 7 days; and PC patients received 40 mg b.i.d. for the first 6 days, then 20 mg b.i.d. for the remaining 23 days. Identical schedules and capsules were used for both the drug and placebo. Compliance with treatment was monitored by a clinical research coordinator, based on pill counts and patient reports. Non-protocol compliance was defined as consuming less than 60% of pills throughout the treatment period, or consuming less than 75% (3 out of 4) of pills in either of the following periods: (i) on the day before surgery and the day of surgery, or (ii) during the first two days following surgery. Intention-to-treat (ITT) definition included all patients who were recruited into the study, regardless of their adherence to the protocol.

### Safety monitoring

On the morning of surgery HR and BP are monitored before administering the highest propranolol dose (80 mg SR b.i.d) under continuous supervision. In any case of HR below 50 bpm or systolic BP below 90 during the treatment phase, treatment is withheld until the symptom is resolved for at least 12 h, and then reinitiated. If bradycardia is not resolved within 12 h (before the next dose), treatment is terminated.

### Randomizations and blinding

Patients were randomized using stratified randomization, ensuring balanced allocation across medical centers. CRC and PC patients were stratified by age (20–59 vs. 60–85), and BC patients were stratified by age (20–49 vs. 50–85). The study was double-blinded; neither patients nor investigators were aware of treatment assignments.

### Study outcomes

(i) Primary outcomes: perioperative safety outcomes, including 16 blood measures sampled on the morning of surgery and the morning after surgery in CRC and PC patients, and on day 30 post-surgery in PC patients; AEs from the initiation of treatments until postoperative day 30, categorized by the Common Terminology Criteria for Adverse Events (CTCAE) and as potentially drug-related vs. not drug-related, including serious adverse events (SAEs). (ii) Secondary outcomes: 8-year DFS and OS in CRC patients. All patients who withdrew or were lost to follow-up were recorded, including reasons for attrition, and were analyzed in an ITT framework.

### Blood samples

Routine blood samples were collected at three time points: (i) 1 day before surgery, (ii) 1 day after surgery, and (iii) for PC patients, approximately 30 days after surgery, after completing the intervention phase, and before starting adjuvant therapy. Samples were analyzed for peripheral immune cells (expressed as a percentage of total white blood cells), including neutrophils, lymphocytes, monocytes, eosinophils, basophils, and neutrophil-to-lymphocyte ratio (NLR). Additional assessments included metabolic parameters such as glucose and creatinine, electrocytes including sodium, potassium, and calcium, liver enzymes SGOT/AST, SGPT/ALT, and AST/ALT ratio, and protein levels including albumin and globulin. Normal range intervals of all these 16 blood measures are standardized and consistent across all study sites.

### AE definition and data collection

AEs were recorded from the first day of treatment, 5 days before surgery, and through 30 postoperative days. Each AE was classified using the CTCAE version 5.0 (US Department of Health and Human Services NIoH and National Cancer Institute, 2017), which provides a grading scale from 1 to 5 to indicate severity. The grades are as follows: Grade 1 - Mild (asymptomatic or mild symptoms, clinical or diagnostic observations only, no intervention required); Grade 2 - Moderate (minimal, local, or noninvasive intervention required); Grade 3 - Severe (medically significant but not immediately life-threatening, hospitalization or prolongation of hospitalization required, disabling); Grade 4 - Life-threatening (urgent intervention required); Grade 5 - Death related to AE. Drug-related AEs were defined as events commonly associated with propranolol and etodolac drugs, or those appearing on a predefined list of known drug-related AEs.

### Statistical analysis

Statistical analysis was conducted using the JASP 0.18.3.0 software. Adverse event differences between the treatment and placebo groups were statistically analyzed using the Fisher’s exact test, or the chi-square test for interaction analyses (rows where expected values of zero were omitted). Cohorts (the different RCTs) were analyzed both separately and in a pooled analysis. For the pooled analysis, relative risk (RR, 95% confidence interval (CI)) and risk difference (RD, 95% CI) are reported. A 2 × 2 repeated-measure ANOVA was employed to assess differences in blood sample indices pre- and post-surgery (within-patient), and to study drug treatment effects (between-patients) for each study separately. Pooled CRC studies data was analyzed using linear mixed models (LMM), with random intercepts for patient ID and a fixed effect for study (COMPIT1 vs. COMPIT2). The model was fitted using restricted maximum likelihood (REML). P-values for treatment effects in blood samples were adjusted for multiple testing using the Benjamini–Hochberg false discovery rate (FDR) procedure, with η^2^ as effect size and the estimated mean difference between groups with 95% CI (Δ [95% CI]). Unadjusted p-values are also presented to highlight potential safety-relevant treatment-related differences. Long-term DFS and OS were analyzed using the Kaplan-Meier curves, adjusted and unadjusted Cox regression models. All analyses were two-sided and conducted based on *a priori* hypotheses.

### Power analysis

Power analysis was conducted using the G*Power 3.1.9.7 software. The power calculation is based on SDs observed in the study samples (post-hoc). Safety analyses were conducted using all available data. For blood indices, employing 9–15 (pancreatic or COMPIT1), 17–20 (COMPIT2), and 27–31 (COMPIT1-2) patients per group provide a power of 0.8 for detecting effects sizes of ∼1.2, ∼0.93, and ∼0.75, respectively. For the study of AEs, a sample size of 72–76 (all RCTs combined) patients per group provides a power of 0.8 to detect a difference corresponding to a rate ratio of ∼1:1.6 (drug:placebo) in a specific AE. When patients’ subgroups are considered separately, this ratio increases to ∼1:2 (COMPIT1-2, 40 per group), ∼1:2.2 (BC, 18–20 per group), and 1:2.3 (PC, 14–16 per group). For long-term cancer outcomes, analyses of DFS and OS are underpowered, with the sample size not sufficient to detect clinically meaningful differences. All analyses assume an alpha level of 0.05 for two-sided differences.

## Results

The primary safety endpoints included AEs and perioperative blood measures. Secondary endpoints were long-term oncological outcomes, 8-year DFS and OS in CRC patients.

### Adverse events

Data from 148 randomized ITT patients from all four RCTs was pooled (drug n = 76; placebo n = 72), and analyzed as a combined dataset, as well as separately for each cancer type (Table 2). Additionally, data from a subset of 121 protocol-compliant patients was similarly analyzed (drug n = 58; placebo n = 63) (Supplementary Table S1). In all four RCTs, AEs were recorded from 5 days before surgery (first day of treatment), and up to 30 days post-surgery. 56.8% (44/148) of ITT patients and 58.6% (71/121) of protocol-compliant patients had no AEs. Of the 148 patients, 64 had at least one AE, and among these, 25 were classified as SAE, and 24 were potentially drug-related.

**TABLE 2:** Safety data for 148 Intent-to-treat patients in the four trials.

All AEs during the safety analysis period	Breast Cancer RCT			Colorectal Cancer RCTs			Pancreatic Cancer RCT			All RCTs				
	Drugs (n = 20)	Placebo (n = 18)	P-val	Drugs (n = 40)	Placebo (n = 40)	P-val	Drugs (n = 16)	Placebo (n = 14)	P-val	Drugs (n = 76)	Placebo (n = 72)	RR (95% CI)	RD (95% CI)	P-val
# of patients with AEs	6	1	0.093	22	19	0.655	8	8	0.730	36 (47%)	28 (39%)	1.22 (0.83 to 1.80)	0.08 (−0.07 to 0.24)	0.323
Potentially drug related	2	0	0.488	9	7	0.781	4	4	1.000	15 (20%)	9 (12%)	1.57 (0.77 to 3.22)	0.07 (−0.03 to 0.18)	0.270
Not drug-related	4	1	0.344	13	12	1.000	4	4	1.000	21 (28%)	19 (26%)	1.05 (0.67 to 1.64)	0.01 (−0.11 to 0.14)	0.310
SAE	2	1	1.000	6	6	1.000	6	7	0.713	14 (18%)	11 (15%)	1.20 (0.61 to 2.37)	0.03 (−0.07 to 0.13)	0.665
AE grade by CTCAE			0.241			0.528			0.357					0.407
0 - No AEs	14	17	0.093	18	21	0.655	8	6	0.730	40 (53%)	44 (61%)	0.86 (0.65 to 1.14)	−0.08 (−0.21 to 0.04)	0.323
1 - Mild	2	0	0.488	5	3	0.712	0	1	0.467	7 (9%)	4 (6%)	1.64 (0.53 to 5.06)	0.04 (−0.04 to 0.12)	0.535
2 - Moderate	2	0	0.488	11	10	1.00	2	0	0.485	15 (20%)	13 (18%)	1.09 (0.61 to 1.96)	0.01 (−0.08 to 0.12)	0.836
3 - Severe	2	1	1.000	4	6	0.737	4	4	1.000	10 (13%)	8 (11%)	1.19 (0.58 to 2.42)	0.02 (−0.06 to 0.10)	0.804
4 - Life-threatening	0	0	1.000	2	0	0.247	2	1	1.000	4 (5%)	1 (1%)	3.78 (0.47 to 30.5)	0.04 (0.00 to 0.09)	0.367
5 - Death	0	0	1.000	0	0	1.000	0	2	0.209	0 (0%)	2 (3%)	0.00 (NA)	−0.03 (−0.08 to 0.03)	0.235
Events per Patient			0.241			0.425			0.504					0.104
0	14	17	0.093	18	21	0.655	8	6	0.730	40	44			0.323
1	3	1	0.606	11	10	1.000	1	2	0.586	15	13			0.836
2	2	0	0.488	5	3	0.712	2	0	0.485	9	3			0.131
3	1	0	1.000	4	1	0.359	2	1	1.000	7	2			0.168
4 and above	0	0	1.000	2	5	0.431	3	5	0.417	5	10			0.177
Most Common AEs														
AE#1 Weakness	1	0	1.000	1	4	0.359	5	4	1.000	7 (9%)	8 (11%)	0.83 (0.34 to 2.03)	−0.02 (−0.09 to 0.05)	0.789
AE#2 Nausea	2	0	0.488	2	4	0.675	3	3	1.000	7 (9%)	7 (10%)	0.95 (0.38 to 2.37)	−0.01 (−0.08 to 0.07)	1.000
AE#3 Abdominal Pain	1	0	1.000	4	5	1.000	1	2	0.586	6 (8%)	10 (14%)	0.57 (0.22 to 1.50)	−0.06 (−0.17 to 0.05)	0.294
AE#4 Surgical Site Infection	0	0	1.000	3	5	0.712	1	1	1.000	4 (5%)	3 (4%)	1.25 (0.30 to 5.26)	0.01 (−0.04 to 0.06)	1.000
AE# 5 Bradycardia	2	0	0.488	5	1	0.201	2	1	1.000	9 (12%)	2 (3%)	4.17 (1.00 to 17.4)	0.10 (0.01 to 0.19)	0.057
AE#6 Bleeding	0	0	1.000	5	2	0.431	2	0	0.485	7 (9%)	2 (3%)	3.29 (0.72 to 15.0)	0.06 (0.01 to 0.12)	0.168

AE, adverse event; SAE; serious adverse event; RR, Risk Ratio; RD, Risk Difference; CI, Confidence Interval. Percentages are rounded to whole numbers. NA indicates calculation not applicable due to zero events.# indicates number (number of patients or medical centers).

In ITT patients, only minor differences between drug and placebo groups were evident, with a trend toward a higher number of bradycardia cases (a drug-related AE) in treated patients within the pooled dataset (9/76 (12%) vs. 2/72 (3%); RR = 4.17 (1.00–17.4), RD = 0.10 (0.01–0.19); p = 0.057). No significant interaction was evident between the drug treatment and the degree of severity of each reported AE. Overall, 5 life-threatening events were recorded, 2 in the CRC patients (drugs) and 3 in the PC patients (2 drugs, 1 placebo), and 2 deaths occurred in PC patients (placebo) (Table 2).

In protocol-compliant patients, minor differences between treatment groups were evident, with only one significant difference in the number of BC patients experiencing AEs (5/19 in the drug group vs. 0/16 in placebo; p = 0.049), with the majority (4 of 5; weakness, nausea, abdominal pain, and surgical site infection) classified as not drug-related AEs. Furthermore, there was a marginally significant difference in the number of bradycardia cases between drugs and placebo groups in CRC patients (5/33 (15%) in the drug group vs. 1/36 (3%) in placebo; p = 0.097) and in the combined analysis (7/58 (12%) vs. 2/63 (3%); RR = 4.00 (1.02–15.7), RD = 0.09 (0.01–0.17); p = 0.085). For all AEs, there was no interaction between treatment and severity of AE (for all p > 0.15). Last, 2 life-threatening events were recorded in drug-treated CRC patients, and 2 deaths occurred in placebo-treated PC patients (non-significant difference) (Supplementary Table S1).

### Perioperative blood biomarkers

Blood samples were taken on the day before and day after the surgery. Data was available only for CRC and PC patients and was first analyzed separately within each population, given differences in baseline profiles. As very few patients were incompliant at this early phase of the treatment, analyses were conducted only on ITT patients. Tables 3, 4 present results from 2 × 2 repeated measure ANOVA, assessing the main effect of surgery (within subjects, before vs. after surgery), the main effect of drug treatment (between subjects), and the interaction between the effects of treatment and of surgery.

**TABLE 3:** Perioperative blood samples were collected and analyzed for colorectal cancer patients from the COMPIT1 and COMPIT2 studies.

Normal Range	Measure	Pre-op Estimated Mean [95% CI]		Post-op Estimated Mean, [95% CI]		Intercept (estimate, t-value, p-value)	Surgery (estimate, t-value, p-value)	Treatment (estimate, t-value, p-value, FDR-adjusted p-value^a^)	Interaction (estimate, t-value, p-value)
		Drugs (n = 31)	Placebo (n = 29–30)	Drugs (n = 34)	Placebo (n = 34)				
1<NLR<2	NLR	7.06 [1.94, 12.19]	9.39 [4.36, 14.41]	11.82 [6.80, 16.83]	15.85 [10.96, 20.73]	11.03, t = 6.53, p < 0.001	2.8, t = 4.21, p < 0.001	−1.58, -, NS, NS	−0.43, -, NS
50%–70%	%Neutrophils^b^	72.21 [68.65, 75.77]	72.04 [68.39, 75.69]	84.07 [80.68, 87.46]	83.30 [79.93, 86.67]	77.90, t = 85.90, p < 0.001	5.78, t = 6.53, p < 0.001	0.23, -, NS, NS	0.15, -, NS
20%–40%	%Lymphocytes^b^	17.71 [14.81, 20.62]	18.59 [15.61, 21.57]	9.38 [6.61, 12.15]	9.38 [6.63, 12.13]	13.77, t = 17.75, p < 0.001	−4.38, t = −6.41, p < 0.001	−0.22, -, NS, NS	0.22, -, NS
2%–8%	%Monocytes	6.87 [5.89, 7.86]	6.85 [5.84, 7.86]	5.98 [5.04, 6.91]	6.48 [5.55, 7.41]	6.54, t = 24.47, p < 0.001	−0.32, -, NS	−0.12, -, NS, NS	−0.13, -, NS
1%–4%	%Eosinophils^b^	2.46 [1.86, 3.06]	1.82 [1.21, 2.44]	0.34 [-0.23, 0.92]	0.47 [-0.1, 1.03]	1.27, t = 8.23, p < 0.001	−0.87, t = −5.9, p < 0.001	0.13, -, NS, NS	−0.19, -, NS
0%–1%	%Basophils^b^	0.45 [0.38, 0.52]	0.5 [0.43, 0.58]	0.17 [0.1, 0.24]	0.17 [0.1, 0.24]	0.32, t = 15.95, p < 0.001	−0.15, t = −9.58, p < 0.001	−0.01, -, NS, NS	0.01, -, NS

		Drugs (n = 26–27)	Placebo (n = 20–22)	Drugs (n = 32–33)	Placebo (n = 30–31)	Intercept (estimate, t-value, p-value)	Surgery (estimate, t-value, p-value)	Treatment (estimate, t-value, p-value, FDR-adjusted p-value^a^)	Interaction (estimate, t-value, p-value)
0.7–1.2 mg/dL	Creatinine mg/dL^b^	0.9 [0.82, 0.98]	0.86 [0.77, 0.94]	0.9 [0.82, 0.97]	0.84 [0.76, 0.92]	0.87, t = 34.67, p < 0.001	−0.01, -, NS	0.02, -, NS, NS	0.003, -, NS
70–100 mg/dL	Glucose mg/dL	112.66 [99.71, 125.62]	104.99 [90.90, 119.08]	138.41 [126.77, 150.5]	127.27 [115.42, 139.13]	120.83, t = 32.68, p < 0.001	−12.00, t = −4.28, p < 0.001	4.7, -, NS, NS	−0.86, -, NS
3.5–5.1 meq/L	Potassium meq/L	4.26 [4.08, 4.43]	4.02 [3.83, 4.21]	4.31 [4.15, 4.46]	4.13 [3.97, 4.29]	4.18, t = 84.32, p < 0.001	−0.04, -, NS	0.1, t = 2.22, p = 0.03, NS	0.01, -, NS
135–145 meq/L	Sodium meq/L	139.03 [138.0, 140.06]	139.28 [138.16, 140.39]	138.0 [137.2, 138.81]	138.36 [137.54, 139.18]	138.67, t = 461.51, p < 0.001	−0.49, t = −2.55, p = 0.015	−0.15, -, NS, NS	−0.027, -, NS
8.6–10.3 mg/dL	Calcium mg/dL	8.84 8.60, 9.09]	8.91 [8.64, 9.2]	8.22 [7.99, 8.44]	8.35 [8.12, 8.58]	8.58, t = 117.31, p < 0.001	−0.3, t = −5.72, p < 0.001	−0.05, -, NS, NS	−0.02, -, NS

		Drugs (n = 24)	Placebo (n = 17–18)	Drugs (n = 28)	Placebo (n = 26–27)	Intercept (estimate, t-value, p-value)	Surgery (estimate, t-value, p-value)	Treatment (estimate, t-value, p-value, FDR-adjusted p-value^a^)	Interaction (estimate, t-value, p-value)
5–38 U/L	SGOT (AST) IU/L^b^	29.8 [22.64, 36.97]	27.84 [19.75, 35.94]	33.40 [26.67, 40.13]	29.18 [22.27, 36.09]	30.06, t = 13.31, p < 0.001	1.23, -, NS	1.55, -, NS, NS	0.56, -, NS
4–41 U/L	SGPT (ALT) IU/L	22.86 [15.24, 30.48]	26.73 [18.90, 34.56]	20.83 [13.35, 28.31]	20.92 [13.46, 28.38]	22.84, t = 8.64, p < 0.001	−1.96, t = −2.70, p = 0.01	−0.99, -, NS, NS	0.95, -, NS
3.4–4.8 g/dL	Albumin g/dL^b^	3.77 [3.57, 3.96]	4.00 [3.78, 4.23]	3.22 [3.03, 3.41]	3.32 [3.13, 3.52]	3.58, t = 56.33, p < 0.001	−0.31, t = −8.73, p < 0.001	−0.08, -, NS, NS	0.03, -, NS
<1	AST/ALT	1.69 [1.33, 2.05]	1.20 [0.82, 1.58]	1.74 [1.39, 2.09]	1.59 [1.24, 1.95]	1.56, t = 12.73, p < 0.001	0.11, t = 2.53, p = 0.016	0.16, -, NS, NS	−0.09, t = −1.99, p = 0.054

		Drugs (n = 17)	Placebo (n = 13)	Drugs (n = 21)	Placebo (n = 21)	Intercept (estimate, t-value, p-value)	Surgery (estimate, t-value, p-value)	Treatment (estimate, t-value, p-value, FDR-adjusted p-value^a^)	Interaction (estimate, t-value, p-value)
2.0–3.5 g/dL	Globulin g/dL	2.62 [2.40, 2.83]	2.75 [2.52, 2.98]	2.31 [2.11, 2.51]	2.36 [2.15, 2.56]	2.51, t = 36.79, p < 0.001	−0.175, t = −4.85, p < 0.001	−0.04, -, NS, NS	0.02, -, NS

CI, Confidence Interval; NS indicates p ≥ 0.1; Borderline p-values (.05 ≤ p < .10) are reported for transparency, but significance threshold is α = 0.05. Estimates are reported with corresponding t-values and p-values from the linear mixed models (LMM). Patient ID was modeled as a random intercept, and study (COMPIT1 vs. COMPIT2) was included as a fixed effect;

^a^ Adjusted using the Benjamini–Hochberg false discovery rate (FDR) method.

^b^ In the measure column indicates parameters for which the fixed effect of study was significant.

**TABLE 4:** Perioperative blood samples were collected and analyzed for 28 pancreatic cancer patients from the BCPC study.

Normal range	Measure	Pre-op mean ± SD		Post-op mean ± SD		Surgery (p-value, effect size)	Treatment (p-value, FDR-adjusted p-value^a^, effect size)	Treatment Effect (Δ [95% CI])	Interaction (p-value, FDR-adjusted p-value^a^, effect size)
		Placebo (n = 13)	Drugs (n = 14–15)	Placebo (n = 13)	Drugs (n = 14–15)				
1<NLR<2	NLR	3.29 ± 3.10	3.09 ± 1.78	7.93 ± 4.71	9.19 ± 5.93	p <0.001, η^2^ = 0.303	NS, NS, -	0.53 [−2.04, 3.1]	NS, NS, -
50%–70%	%Neutrophils	60.60 ± 15.12	63.82 ± 13.65	77.62 ± 16.14	78.95 ± 9.44	p <0.001, η^2^ = 0.268	NS, NS, -	2.27 [−6.9, 11.44]	NS, NS, -
20%–40%	%Lymphocytes	29.15 ± 13.49	24.05 ± 12.22	14.49 ± 12.30	12.16 ± 6.55	p <0.001, η^2^ = 0.262	NS, NS, -	−3.72 [−11.37, 3.93]	NS, NS, -
2%–8%	%Monocytes	8.25 ± 3.93	8.59 ± 3.19	6.95 ± 3.18	7.38 ± 3.16	p = 0.086, η^2^ = 0.036	NS, NS, -	0.38 [−1.81, 2.57]	NS, NS, -
1%–4%	%Eosinophils	1.34 ± 1.03	2.98 ± 2.79	0.37 ± 0.45	1.17 ± 1.28	p = 0.004, η^2^ = 0.136	p = 0.015, p^a^ = NS, η^2^ = 0.106	1.22 [0.26, 2.18]	NS, NS, -
0%–1%	%Basophils	0.67 ± 0.37	0.55 ± 0.33	0.55 ± 1.02	0.33 ± 0.24	NS, -	NS, NS, -	−0.17 [−0.46, 0.12]	NS, NS, -

		Placebo (n = 11–13)	Drugs (n = 13–15)	Placebo (n = 11–13)	Drugs (n = 13–15)				
0.7–1.2 mg/dL	Creatinine mg/dL	0.70 ± 0.25	0.80 ± 0.23	0.68 ± 0.23	0.83 ± 0.30	NS, -	NS, NS, -	0.12 [−0.05, 0.3]	NS, NS, -
70–100 mg/dL	Glucose mg/dL	131.00 ± 32.35	124.33 ± 30.36	139.47 ± 21.87	140.46 ± 40.44	p = 0.091, η^2^ = 0.037	NS, NS, -	−2.83 [−23.42, 17.76]	NS, NS, -
3.5–5.1 meq/L	Potassium meq/L	4.32 ± 0.70	4.47 ± 0.47	4.21 ± 0.36	4.29 ± 0.46	NS, -	NS, NS, -	0.11 [−0.19, 0.42]	NS, NS, -
135–145 meq/L	Sodium meq/L	139.15 ± 3.93	139.07 ± 1.10	138.77 ± 2.92	139.27 ± 3.55	NS, -	NS, NS, -	0.2 [−1.87, 2.28]	NS, NS, -
8.6–10.3 mg/dL	Calcium mg/dL	9.28 ± 0.39	9.47 ± 0.59	8.61 ± 0.47	8.72 ± 0.56	p <0.001, η^2^ = 0.332	NS, NS, -	0.15 [−0.15, 0.44]	NS, NS, -
5–38 U/L	SGOT (AST) IU/L	66.75 ± 76.26	46.77 ± 67.69	127.25 ± 99.09	151.00 ± 126.81	p = 0.002, η^2^ = 0.166	NS, NS, -	1.88 [−60.73, 64.5]	NS, NS, -
4–41 U/L	SGPT (ALT) IU/L	70.91 ± 119.82	54.69 ± 71.75	100.92 ± 83.87	135.15 ± 119.12	p = 0.008, η^2^ = 0.074	NS, NS, -	9.01 [−64.73, 82.74]	NS, NS, -
<1	AST/ALT	1.33 ± 0.49	1.00 ± 0.41	1.50 ± 1.09	1.08 ± 0.28	NS, -	NS, NS, -	−0.38 [−0.84, 0.08]	NS, NS, -
3.4–4.8 g/dL	Albumin g/dL	3.66 ± 0.56	3.66 ± 0.59	3.03 ± 0.43	3.04 ± 0.53	p <0.001, η^2^ = 0.270	NS, NS, -	0.01 [−0.35, 0.36]	NS, NS, -

		Placebo (n = 9)	Drugs (n = 12)	Placebo (n = 9)	Drugs (n = 12)				
2.0–3.5 g/dL	Globulin g/dL	2.81 ± 0.58	2.82 ± 0.55	2.08 ± 0.49	2.07 ± 0.34	p <0.001, η^2^ = 0.375	NS, NS, -	0.001 [−0.35, 0.36]	NS, NS, -

SD = Standard Deviation. NS indicates p ≥ 0.1; Borderline p-values (.05 ≤ p <0.10) are reported for transparency, but significance threshold is α = 0.05.

^a^ Adjusted using the Benjamini–Hochberg false discovery rate (FDR) method. Effect sizes are reported as partial eta squared (η2) for repeated measures ANOVA. CI = Confidence Interval. Treatment Effect (Δ [95% CI]) represents the model-based estimate of the mean difference (Drugs − Placebo) with 95% CIs, reported regardless of statistical significance. Normal range intervals of all 16 blood measures are standardized and consistent across all study sites. Two samples were excluded from analysis of SGOT (AST) and SGPT (ALT) due to outlier values (>3 SD from the mean).

### Colorectal cancer RCTs

Data from CRC patients, COMPIT1 (n = 21) and COMPIT2 (n = 37) studies, was analyzed as a pooled dataset with study as a fixed effect and patient ID as a random intercept (Table 3). Separate analyses of the two RCTs are reported in Supplementary Tables S2, S3.

Surgery significantly affected 11 of 16 blood biomarkers, with 6 indices deviating from normal ranges postoperatively. Surgery was associated with significant post-operatively decrease in %basophils (−0.15, t = −9.58, p < 0.001), %lymphocytes (−4.38, t = −6.41, p < 0.001) and %eosinophils (−0.87, t = −5.9, p < 0.001), and in levels of albumin (−0.31, t = −8.73, p < 0.001) and globulin (−0.175, t = −4.85, p < 0.001). SGPT decreased post-operatively (−1.96, t = −2.70, p = 0.01), while AST/ALT ratio increased (0.11, t = 2.53, p = 0.016). Last, calcium was decreased following surgery (−0.3, t = −5.72, p < 0.001). These effects of surgery have been previously reported, but their relation to the drug treatment was hardly studied.

Drug treatment was associated with higher potassium levels (0.1, t = 2.22, p = 0.03), but this outcome did not remain significant after correction for multiple comparisons. No interactions were found between the effects of drug treatment and surgery, indicating that the treatment did not significantly worsen or improve the effects of surgery.

### Pancreatic cancer RCT

Blood sample data of PC patients in the BCPC study (n = 28) was analyzed. Significant effects of surgery were found in 9 of the 16 blood biomarkers, with 7 of these being outside the normal range, including peripheral immune cells (%neutrophils, %lymphocytes, and %eosinophils), as well as SGOT, SGPT, and albumin levels (Table 4). Importantly, the drug-treated group had significantly higher %eosinophils compared to placebo both pre- and post-operatively (Δ [95% CI] = 1.22 [0.26, 2.18], η^2^ = 0.106), although this effect did not remain significant after correction for multiple comparisons (p = 0.015, FDR-adjusted p = NS, η^2^ = 0.106).

### Long-term outcomes of eight-year follow-up

#### Colorectal cancer RCT (COMPIT1)

This study is not powered to the following outcomes, which are conducted as exploratory analyses.

**FIGURE 1:**
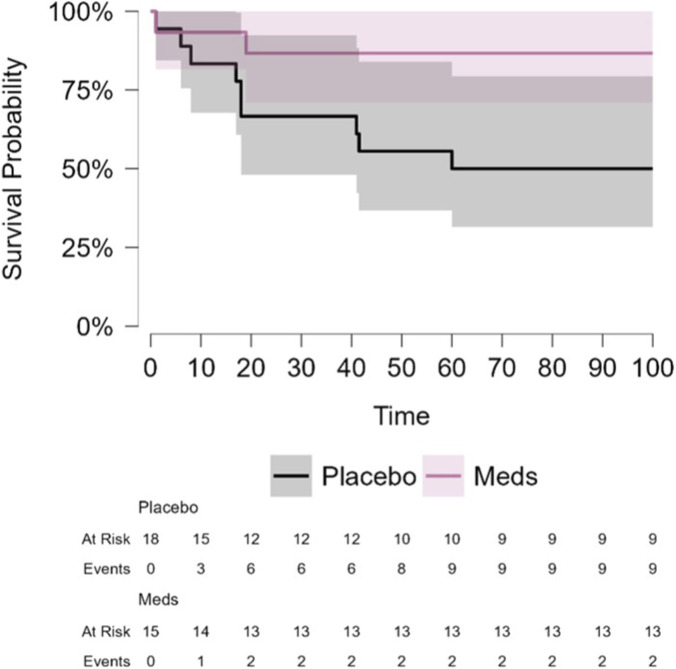
COMPIT 1- Kaplan-Meier curve for 8-year DFS in the ITT population. Time in months. Colorectal cancer patients (COMPIT1 study, intent-to-treat population) treated with perioperative propranolol and etodolac (Meds/drug) had significantly fewer recurrence compared to placebo (2/15 vs. 9/18; p = 0.034). HR for drug vs. placebo from unadjusted Cox regression model: 0.22 (95% CI, 0.05–1.02).

DFS: Drug-treated patients exhibited a significantly lower recurrence rate in both ITT and protocol-compliant groups. In ITT patients (n = 33), 2 of 15 drug-treated and 9 of 18 placebo-treated patients experienced recurrence (p = 0.034, log-rank test) (Figure 1). Unadjusted Cox regression analysis showed a hazard ratio (HR) of 0.22 (95% CI, 0.05–1.02, p = 0.054). Adjusted Cox model, with age, disease stage, and body mass index (BMI) as covariates, yielded a HR of 0.15 (95% CI, 0.03–0.81, p = 0.027) (Supplementary Table S5). In protocol-compliant patients (n = 28), 0 of 11 drug-treated and 8 of 17 placebo-treated patients experienced recurrence (p = 0.01), and HR could not be estimated due to zero events in the treatment group (Supplementary Figure S1; Supplementary Table S6).

**FIGURE 2:**
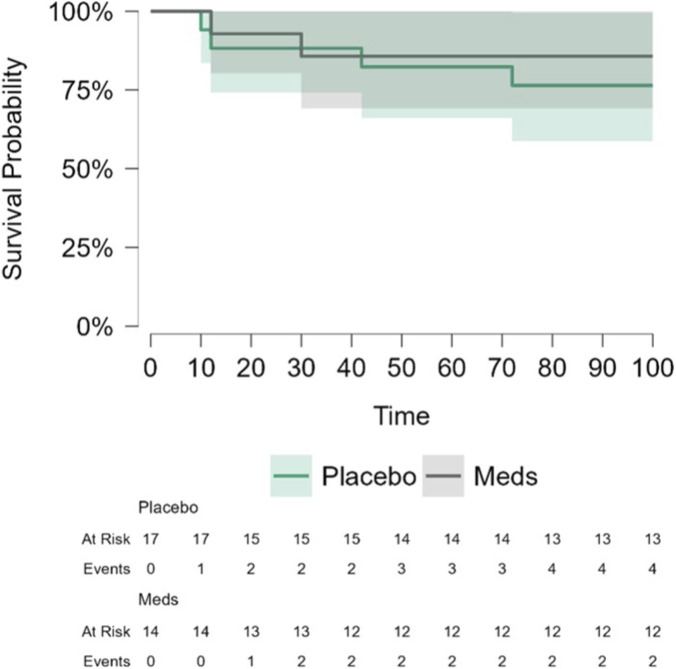
COMPIT 1- Kaplan-Meier curve for 8-year OS in the ITT population. Time in months. Death rate in drug-treated group was lower compared to placebo (2/14 vs. 4/17 in placebo (p = 0.534); not statistically significant. HR for drug vs. placebo from unadjusted Cox regression model: 0.64 (95% CI, 0.1–4.0).

OS: In ITT (n = 31) and protocol-compliant (n = 28) groups, the death rate was non-significantly lower in drug-treated patients compared to placebo-treated patients (2 of 14 vs. 4 of 17; p = 0.534, and 0 of 11 vs. 4 of 17; p = 0.091, respectively) (Figure 2; Supplementary Figure S2). Unadjusted Cox regression analysis showed a HR of 0.59 (95% CI, 0.11–3.22, p = 0.54), and the adjusted model (with age, disease stage, and BMI as covariates) showed a HR of 0.64 (95% CI, 0.1–4.0, p = 0.64). For protocol-compliant patients, HR could not be estimated due to zero events in the treatment group (Supplementary Tables S7-S8).

## Discussion

This study assessed the safety of a perioperative treatment schedule with propranolol and etodolac for cancer patients in four RCTs (n = 148). It revealed a good safety profile. The treatment was identical in all RCTs in its first 7 days, starting 5 days preoperatively until postoperative day 1, covering the highest doses in all RCTs. Safety AEs were assessed separately by cancer type, and in a pooled dataset. Blood biomarkers were analyzed separately and pooled only for CRC studies, given their identical protocols.

AEs, from the initiation of treatments until postoperative day 30, were categorized by: (i) 1-5 severity score based on CTCAE classification, (ii) as potentially drug-related vs. not drug-related, and (iii) by the specific six most common AEs (e.g., bradycardia, nausea). There was no significant effect of the drug treatment, whether analyzed separately in each RCT or combined across all four RCTs, regarding any of these categories, except for the non-severe AE of bradycardia, which was increased by the drug treatment in the pooled patients’ population. Importantly within the SAE categories (severe, life-threatening, and mortality), drug- and placebo-treatment patients were similarly distributed, with 2 cases of mortality in placebo-treated pancreatic patients.

Bradycardia is an expected physiological response to propranolol, is not a severe or life-threatening event in patients with no contraindication to the treatment (i.e., patients recruited to these RCTs), and is easily resolved, as per our protocol (see Methods). In future studies propranolol doses can be personalized, decreased or increased, based on response criteria to ensure drug efficacy while preventing bradycardia. Indeed, in BC patients, Hiller et al. reported significant individual variations in HR and BP response to 20 mg b.i.d propranolol before surgery, and increased the doses to 40 mg b.i.d in patients that did not exhibit significant responses (Hiller et al., 2020).

Overall, based on a total of 64 of 148 ITT patients, and 50 of 122 protocol-compliant patients experiencing at least one AE, only bradycardia was substantially affected, with higher rates in the drug treatment group (ITT: 12% vs. 3%, p = 0.057; protocol-compliant: 9% vs. 3%, p = 0.085). Although not statistically significant, bleeding events were also more frequent in the drug treatment group (ITT: 7/76 (9%) vs. 2/72 (3%), p = 0.168; protocol-compliant: 6/58 (10%) vs. 2/63 (3%), p = 0.151), indicating the need for further investigation in larger cohorts. Importantly, a recent RCT in PC patients (the PROSPER trial) tested a nearly identical perioperative regimen and found no safety concerns (Hüttner et al., 2025). The trial reported fewer SAEs in the treatment group and showed promising trends in DFS. These findings further support the safety of this combined drug approach and the rationale for larger trials.

The 16 blood biomarkers were a-priori selected for analysis, based on a comprehensive literature review (Benish et al., 2008; McDonald et al., 2001; Brown et al., 2006; Garden et al., 1990; Ghebeh et al., 2022; Hadadi et al., 2022; Hai et al., 2018; Kang et al., 2014; Klinck et al., 2015; Li et al., 2018; Rubinkiewicz et al., 2019; Shiratori et al., 2022; Ye et al., 2020; Zhang et al., 2022), to assess immune and medical status, treatment safety and patients’ health. These indices were compared (i) between drug- and placebo-treated patients, and (ii) within-patient between the morning before surgery and the morning after surgery.

Overall, surgery significantly affected most of these indices, increasing or decreasing their levels beyond normal ranges. The drug treatment affected only a few indices. Eosinophils level was significantly higher in the drug-treated group, before and after surgery in the pancreatic patients, preventing the postoperative reduction (to below standard levels) observed in the placebo group. Higher preoperative eosinophils levels in drug-treated patients may suggest a positive prognostic outcome, as studies suggested that eosinophils may play an anti-tumorigenic role in various cancers, including CRC (Varricchi et al., 2018). Additionally, higher peripheral eosinophil levels in lung cancer patients treated with immunotherapy was suggested to predict better outcomes (Alves et al., 2021), and in BC patients, higher eosinophil levels was significantly correlated with response to therapy, and improved DFS after therapy (Ghebeh et al., 2022). In CRC patients, the drug-treated group showed significantly lower postoperative albumin and calcium levels, both only slightly below the normal range. These decreases suggest mildly negative effects, potentially reflecting systemic stress or inflammatory response (Labgaa et al., 2017). However, these changes were likely not clinically significant, given the overall safety profile and absence of stress-inflammatory related adverse events. Potassium levels were higher in the drug-treated group, although within normal limits. These higher levels may reflect improved electrolyte regulation and are interpreted as neutral to mildly favorable (Yang et al., 2019). Importantly, after correction for multiple testing, none of the observed differences in blood indices remained statistically significant. This suggests that these findings should be interpreted with caution and considered exploratory until validated in larger studies. Overall, these findings support the safety of the treatment and suggest potential immune benefits. Our main observation is that surgery affected the studied safety biomarkers far more profoundly than the drug treatment, and given the AE findings, the latter are likely clinically insignificant.

We are currently only able to report long-term DFS and OS outcomes, as a secondary outcome, for the first completed CRC trial, which is limited by being statistically underpowered for these indices. The PC and the second CRC trials are still ongoing, and long-term follow-up data of BC trial is unavailable. Outcomes are reassuring in terms of treatment safety and suggest long-term advantages. Specifically, in 8-year DFS of CRC patients, drug treatment showed a significant advantage, with less patients experiencing disease recurrence, compared to placebo (2 of 15 vs. 9 of 18, ITT; p = 0.034), similar to the previously reported 5-year follow-up in this trial (Ricon-Becker et al., 2023). We hypothesize that the drug treatment may be more effective in eliminating or preventing the progression of single tumor cells and micro-metastases, rather than larger metastases. If so, drug impact may only become apparent after extended follow-up periods, when some micro-metastases progress to detectable lesions. Indeed, in our translational studies, long-term benefits of the treatment were only evident in the latter half of the follow-up period (Glasner et al., 2010).

Importantly, some concerns have been raised regarding the perioperative use of β-blockers and COX inhibitors, though not specifically regarding non-selective β-blockers like propranolol or semi-selective COX-2 inhibitors like etodolac, as used in our studies. Historically, the perioperative use of β-blockers was recommended by the European Society of Cardiologists (ESC), American College of Cardiology (ACC), and the American Heart Association (AHA) (Priebe, 2014), for patients at risk of postoperative cardiac events. However, this recommendation was reassessed in 2008 following the POISE study involving 8,351 subjects (Group et al., 2008). This study found that while 100 mg of extended-release metoprolol, a β_1_-selective antagonist, reduced myocardial infarction (4.2% vs. 5.7%), it was also linked to higher postoperative mortality rate (3.1% vs. 2.3%) mainly due to hypotension and stroke. The adverse outcomes were attributed to the moderate to high dosing, and the initiation of treatment on the day of surgery without prior dose escalation. However, a subsequent large cohort study with 44,092 patients in 2013 suggested that the increased stroke risk was specific to metoprolol, not other β_1_-blockers (Ashes et al., 2013). Furthermore, in 2014 the ACC/AHA and ESC revised their guidelines, recommending that β-blockers should be initiated preoperatively to assess safety and tolerability, cautioning against starting therapy on the day of surgery (Kristensen and Knuuti, 2014; Fleisher et al., 2014). The ESC specifically recommend starting β-blockers at least 2 days before surgery and continuing postoperatively (Kristensen and Knuuti, 2014), as indeed implemented in our four RCTs, with additional safety measures (see Methods).

Recent retrospective evidence indicates a safe profile and protective effect of perioperative non-selective β-blockers (such as propranolol) against cancer progression. Specifically, in a cohort of 3,844 ovarian cancer patients, it led to a significantly higher survival rate compared to non-users (80% vs. 69% at 2 years postoperatively), and such survival advantage was maintained for at least 8 years (Spilsbury et al., 2023). Similarly, in prostate cancer, it was significantly associated with reduced risk of cancer recurrence among 11,117 patients (Sivanesan et al., 2022). Last, research indicates that the use of non-selective β-blockers in hospitalized cirrhosis patients was found safe and protective against infection during hospitalization (Tittanegro et al., 2023).

Regarding COX-2 inhibitors, concerns about perioperative use of these drugs and cardiovascular events, bleeding, or bone healing have been raised. However, a protocol of the ERAS (Enhanced Recovery After Surgery) society concludes that current evidence does not justify avoiding NSAIDs during the short perioperative period in patients with low cardiovascular risk (Feldheiser et al., 2016), as evidence is inconclusive and studies have not shown an increase in bleeding, anastomotic leakage, cardiovascular risk, or delayed bone healing with perioperative COX-2 inhibitors (Feldheiser et al., 2016; Buvanendran et al., 2003; Teerawattananon et al., 2017). However, in patients at high risk for heart failure, myocardial infarction, or stroke, which are excluded from our RCTs, NSAIDs should be used at low doses and for short duration (Schmidt et al., 2016). Finally, while disturbances to tissue healing should be a concern, recent animal studies have reported no adverse effects from either propranolol, etodolac, or both on colon anastomosis, skin, and muscle healing, with some beneficial effects reported (Benjamin et al., 2010; Hazut et al., 2011).

By and large, our stringent exclusion criteria exclude approximately 60% of colorectal and pancreatic patients. If ongoing RCTs provide conclusive evidence of improvements in long-term outcomes, physicians might consider substituting several exclusion criteria with more rigorous monitoring of patients with known contraindications to the drugs. For example, patients with mild cardiac conditions or those on medications that currently exclude them from participation might be included, under stricter surveillance (e.g., frequent cardiovascular assessments), or while adjusting drug dosages.

Several limitations should be considered when interpreting the findings. The major limitation is the relatively small sample size of 148 patients from 4 different RCTs with different cancer types. Another limitation for AEs analysis is the limited follow-up period of 30 postoperative days. Larger patient cohort and extended follow-up may yield a more precise estimation of risks and benefits of the drug treatment, regarding each AE, and specifically important for AE of low frequency and high severity. For blood biomarkers, since no baseline measures were collected prior to drug administration (and despite group randomization), more subtle impacts of the drugs may have not been detected, and baseline levels cannot be used as covariate. It should be stressed though that the current study is powered for relatively small effects sizes of 0.75–1.2, in the different cohorts. Future studies should collect baseline blood measures in larger cohorts to assess whether certain pre-treatment parameters constitute risk factors for this drug treatment. Last, it is worth noting that this safety assessment was conducted only in patients who met the inclusion and exclusion criteria for these RCTs (40% or less). Thus, this safety analysis is only applicable to patients with these specific characteristics.

This is the first systematic assessment of a safety profile of perioperative combined propranolol and etodolac treatment in cancer patients. In our completed BC and CRC trials, the treatment led to favorable changes in the excised tumors, including (i) decreased EMT, (ii) decreased tumor-infiltrating CD14^+^ monocytes, (iii) favorably modified GATA, STAT, and EGR transcription factor activity, and (iv) increased tumor-infiltrating Natural Killer cells in CRC patients (Haldar et al., 2020; Shaashua et al., 2017; Hiller et al., 2020). Additionally, chronic use of either COX inhibitors or β-adrenergic blockers has been retrospectively associated with improved cancer outcomes in several types of cancer and large patient populations (Horowitz et al., 2015; Hiller et al., 2018; Ricon et al., 2019; Yap et al., 2018). Our preclinical studies also indicated the partial efficacy of each drug alone, while suggesting additive or synergistic effects of the combined treatment. Thus, a single-drug clinical regimen may also be effective. Taken together, while hypothetical risks of the combined use of propranolol and etodolac exist, they appear low and manageable when weighed against the accumulating evidence for their potential benefits reported by translational, epidemiological, and our completed RCTs (Haldar et al., 2020; Haldar et al., 2018; Shaashua et al., 2017). Finally, the current cumulative safety assessment suggests a favorable benefit-risk profile and highlights the need for adequately powered trials with long-term follow-up to validate these findings and further assess potential rare or delayed risks.

## Data Availability

The raw data supporting the conclusions of this article will be made available by the authors, without undue reservation.
