# Supplementary material for: Favorable safety outcomes of a perioperative propranolol and etodolac regimen in cancer patients in four randomized controlled trials
Source: Front Pharmacol. 2026 Apr 20;17:1823113. doi: 10.3389/fphar.2026.1823113 (PMC13136142; doi:10.3389/fphar.2026.1823113)
Supplement: Supplementary file 1 [file Supplementaryfile1.docx]

**Results**

**Adverse events:**

| **Supplementary Table S1. Safety data for 121 protocol-compliant patients in the four studies** | | | | | | | | | | | | | | |
| --- | --- | --- | --- | --- | --- | --- | --- | --- | --- | --- | --- | --- | --- | --- |
| **All AEs during the safety analysis period** | Breast Cancer RCT | | | Colorectal Cancer RCTs | | | Pancreatic Cancer RCT | | | All RCTs | | | | |
|  | Drugs  (n=19) | Placebo  (n=16) | P-Val | Drugs  (n=33) | Placebo  (n=36) | P-Val | Drugs  (n=6) | Placebo  (n=11) | P-Val | Drugs  (n=58) | Placebo  (n=63) | RR (95% CI) | RD (95% CI) | P-Val |
| # Patients with AEs | 5 | 0 | 0.049 | 20 | 17 | 0.366 | 3 | 5 | 1.000 | 28 (48%) | 22 (35%) | 1.36 (0.88–2.09) | 0.13 (–0.03–0.28) | 0.145 |
| Potentially related | 1 | 0 | 1.000 | 8 | 7 | 0.772 | 2 | 2 | 0.584 | 11 (19%) | 9 (14%) | 1.38 (0.66–2.88) | 0.05 (–0.06–0.16) | 0.625 |
| Not drug-related | 4 | 0 | 0.109 | 12 | 10 | 0.606 | 1 | 3 | 1.000 | 17 (29%) | 13 (21%) | 1.38 (0.78–2.45) | 0.08 (–0.03–0.19) | 0.298 |
| SAE | 0 | 0 | NA | 6 | 6 | 1.000 | 2 | 4 | 1.000 | 9 (16%) | 9 (14%) | 1.13 (0.53–2.42) | 0.02 (–0.10–0.14) | 1.000 |
| **AE grade by CTCAE** |  | | **0.178** |  | | **0.508** |  | | **0.643** |  | |  |  | **0.249** |
| 0 - No AEs | 14 | 16 | 0.049 | 13 | 19 | 0.336 | 3 | 6 | 1.000 | 30 (52%) | 41 (65%) | 1.64 (0.53–5.06) | 0.04 (–0.04–0.12) | 0.145 |
| 1 - Mild | 2 | 0 | 0.489 | 4 | 3 | 0.702 | 0 | 0 | 1.000 | 6 (10%) | 4 (6%) | 1.55 (0.78–3.07) | 0.08 (–0.03–0.19) | 0.518 |
| 2 - Moderate | 2 | 0 | 0.489 | 10 | 9 | 0.788 | 1 | 1 | 1.000 | 13 (22%) | 9 (14%) | 1.07 (0.46–2.50) | 0.01 (–0.09–0.11) | 0.346 |
| 3 - Severe | 1 | 0 | 1.000 | 4 | 5 | 1.000 | 2 | 2 | 0.584 | 7 (12%) | 7 (11%) | NA | 0.03 (0.00–0.09) | 1.000 |
| 4 - Life-threatening | 0 | 0 | 1.000 | 2 | 0 | 0.225 | 0 | 0 | 1.000 | 2 (3%) | 0 (0%) | 0.00 (NA) | –0.03 (–0.08–0.03) | 0.228 |
| 5 - Death | 0 | 0 | 1.000 | 0 | 0 | 1.000 | 0 | 2 | 0.515 | 0 (0%) | 2 (3%) | 1.64 (0.53–5.06) | 0.04 (–0.04–0.12) | 0.497 |
| **Events per Patient** |  | | **0.178** |  | | **0.453** |  | | **0.597** |  | |  |  | **0.131** |
| 0 | 14 | 16 | 0.049 | 13 | 19 | 0.336 | 3 | 6 | 1.000 | 30 | 41 |  |  | 0. 145 |
| 1 | 3 | 0 | 0.234 | 10 | 9 | 0.788 | 0 | 1 | 1.000 | 13 | 10 |  |  | 0.487 |
| 2 | 1 | 0 | 1.000 | 4 | 3 | 0.702 | 1 | 0 | 0.353 | 6 | 3 |  |  | 0.309 |
| 3 | 1 | 0 | 1.000 | 4 | 1 | 0.186 | 1 | 1 | 1.000 | 6 | 2 |  |  | 0.151 |
| 4 and above | 0 | 0 | 1.000 | 2 | 4 | 0.675 | 1 | 3 | 1.000 | 3 | 8 |  |  | 0.209 |
| Most Common AEs | | | | | | | | | | | | | | |
| AE#1 Weakness | 1 | 0 | 1.000 | 1 | 3 | 0.615 | 2 | 3 | 1.000 | 4 (7%) | 6 (10%) | 0.70 (0.25–1.98) | –0.03 (–0.10–0.04) | 0.745 |
| AE#2 Nausea | 2 | 0 | 0.489 | 1 | 4 | 0.359 | 2 | 2 | 0.584 | 5 (9%) | 6 (10%) | 0.90 (0.37–2.21) | –0.01 (–0.07–0.06) | 1.000 |
| AE#3 Abdominal Pain | 1 | 0 | 1.000 | 5 | 4 | 0.728 | 0 | 0 | 1.000 | 5 (9%) | 8 (13%) | 0.72 (0.29–1.78) | –0.04 (–0.12–0.03) | 0.564 |
| AE#4 Surgical Site Infection | 0 | 0 | 1.000 | 2 | 5 | 0.431 | 1 | 2 | 1.000 | 4 (7%) | 3 (5%) | 1.38 (0.35–5.45) | 0.02 (–0.04–0.07) | 0.709 |
| AE# 5 Bradycardia | 1 | 0 | 1.000 | 5 | 1 | 0.097 | 1 | 1 | 1.000 | 7 (12%) | 2 (3%) | 4.00 (1.02–15.7) | 0.09 (0.01–0.17) | 0.085 |
| AE#6 Bleeding | 0 | 0 | 1.000 | 5 | 2 | 0.247 | 1 | 0 | 0.353 | 6 (10%) | 2 (3%) | 3.33 (0.74–14.9) | 0.07 (0.01–0.13) | 0.151 |

AE = adverse event; SAE = serious adverse event; RR = Risk Ratio; RD = Risk Difference; CI = Confidence Interval. Percentages are rounded to whole numbers. NA indicates calculation not applicable due to zero events.

**Perioperative blood biomarkers**:

**Colorectal cancer RCTs- COMPIT1**

Blood sample data of CRC patients in the COMPIT2 study (n=21) was analyzed. Significant surgery effects were found in 3 of 16 blood biomarkers (%monocytes, %eosinophils, and %basophils) (Table S2). Also, potassium levels were significantly higher in the drug-treated group compared to placebo (p=.05, η²=.244). Calcium and albumin levels were significantly lower in the drug-treated group (p=.036, η²=.274; p=.03, η²=.345, respectively). The AST/ALT ratio was also significantly higher in the drug-treated group (p=.042, η²=.333). %Lymphocytes and %basophils, although not significantly, tended to be lower in the drug-treated group compared to placebo (p=.058, η²=.116; p=.072, η²=.077). Globulin levels showed a trend toward being lower in the drug-treated group compared to placebo (p=.066, η²=.256).

| **Supplementary Table S2. Perioperative blood samples were collected and analyzed for colorectal cancer patients from the COMPIT1 study** | | | | | | | | | |
| --- | --- | --- | --- | --- | --- | --- | --- | --- | --- |
| **Normal Range** | **Measure** | **Pre-op Mean ± SD** | | **Post-op Mean ± SD** | | **Surgery**  **(p-value, effect size)** | **Treatment**  **(p-value, FDR-adjusted p-value^a^, effect size)** | **Treatment Effect**  **(Δ [95% CI])** | **Interaction**  **(p-value, FDR-adjusted p-value^a^, effect size)** |
|  |  | **Drugs**  (n=11) | **Placebo**  (n=10) | **Drugs**  (n=11) | **Placebo**  (n=10) |  |  |  |  |
| 1<NLR<2 | NLR | 13.20±10.81 | 11.28±8.38 | 13.34±7.39 | 9.04±5.43 | NS, - | NS, NS, - | 3.11 [−3.02, 9.24] | NS, NS, - |
| 50-70% | %Neutrophils | 82.41±10.31 | 80.07±12.92 | 84.32±5.40 | 80.28±5.91 | NS, - | NS, NS, - | 3.19 [−3.14, 9.52] | NS, NS, - |
| 20-40% | %Lymphocytes | 8.85±4.46 | 14.17±11.19 | 7.99±4.91 | 12.23±5.08 | NS, - | p=.058, p^a^=.165, η²=.116 | −4.78 [−9.74, 0.18] | NS, NS, - |
| 2-8% | %Monocytes | 6.04±2.91 | 4.69±2.33 | 7.36±2.13 | 6.84±1.94 | p=.017, η²=.124 | NS, NS, - | 0.93 [−0.72, 2.59] | NS, NS, - |
| 1-4% | %Eosinophils | 2.41±4.23 | 0.71±0.63 | 0.35±0.36 | 0.26±0.41 | p=.08, η²=.076 | NS, NS, - | 0.9 [−0.55, 2.34] | NS, NS, - |
| 0-1% | %Basophils | 0.27±0.19 | 0.37±0.14 | 0.13±0.13 | 0.22±0.15 | p=.004, η²=.187 | p=.072, p^a^=.165, η²=.077 | −0.09 [−0.2, 0.01] | NS, NS, - |
|  |  | **Drugs**  (n=7)  27) | **Placebo**  (n=3-4)  20) | **Drugs**  (n=7)  27) | **Placebo**  (n=3-4)  20) |  |  |  |  |
| 0.7-1.2 mg/dL | Creatinine mg/dl | 1.01 ±0.13 | 1.05±0.14 | 1.02±0.20 | 1.18±0.34 | NS, - | NS, NS, - | −0.1 [−6.53, 55.92] | NS, NS, - |
| 70-100 mg/dL | Glucose mg/dl | 126.57±36.56 | 104.75±31.19 | 122.57±25.39 | 95.00±24.01 | NS, - | NS, NS, - | 24.7 [−3.02, 9.24] | NS, NS, - |
| 3.5-5.1 meq/L | Potassium meq/l | 4.66±0.69 | 4.00±0.36 | 4.64±0.48 | 4.2±0.18 | NS, - | p=.05, p^a^=.165, η²=.244 | 0.55 [−0.001, 1.1] | NS, NS, - |
| 135-145 meq/l | Sodium meq/l | 139.00±1.83 | 137.75±1.71 | 137.57±2.37 | 137.75±2.50 | NS, - | NS, NS, - | 0.54 [−1.15, 2.22] | NS, NS, - |
| 8.6-10.3 mg/dL | Calcium mg/dl | 8.34+0.87 | 9.40+0.53 | 8.13+0.48 | 8.90+1.04 | NS, - | p=.036, p^a^=.165, η²=.274 | −0.91 [−1.75, -0.08] | NS, NS, - |
| 5-38 U/L | SGOT (AST) IU/l | 31.14±12.88 | 28.67±18.50 | 42.14±29.58 | 35.00±20.88 | NS, - | NS, NS, - | 4.81 [−24.0, -33.62] | NS, NS, - |
| 4-41 U/L | SGPT (ALT) IU/l | 22.29±14.48 | 27.40±28.39 | 24.42±16.52 | 20.77±12.87 | NS, - | NS, NS, - | −9.02 [−38.75, 20.7] | NS, NS, - |
| 3.4-4.8 g/dl | Albumin g/dl | 3.29±0.61 | 4.17±0.55 | 2.96±0.33 | 3.73±0.83 | NS, - | p=.03, p^a^=.165, η²=.345 | −0.83 [−1.55, -0.1] | NS, NS, - |
| 2.0-3.5 g/dL | Globulin g/dl | 2.33±0.40 | 2.93±0.60 | 2.24±0.32 | 2.60±0.36 | NS, - | p=.066, p^a^=.165, η²=.256 | −0.48 [−1.0, 0.04] | NS, NS, - |
| <1 | AST/ALT | 1.71±0.49 | 1.00±0.00 | 1.86±0.38 | 1.33±0.58 | NS, - | p=.042, p^a^=.165, η²=.333 | 0.62 [0.03, 1.21] | NS, NS, - |

SD = Standard Deviation. NS indicates p ≥ 0.1; Borderline p-values (.05 ≤ p < .10) are reported for transparency, but significance threshold is α = 0.05. ^a^Adjusted using the Benjamini–Hochberg false discovery rate (FDR) method. Effect sizes are reported as partial eta squared (η²) for repeated measures ANOVA. CI = Confidence Interval. Treatment Effect (Δ [95% CI]) represents the model-based estimate of the mean difference (Drugs − Placebo) with 95% CIs, reported regardless of statistical significance.

**Colorectal cancer RCTs- COMPIT2**

Blood sample data of CRC patients in the COMPIT2 study (n=37) was analyzed. Significant surgery effects were found in 12 of 16 blood biomarkers, with 8 of these being outside the normal range, including peripheral immune cells (%neutrophils, %lymphocytes, %monocytes and %eosinophils), as well as glucose, calcium, albumin levels (Table S3). However, no significant differences in these biomarkers were observed between the treatment groups. NLR increased post-operatively (p<.001, η²=.326) and tended to be lower in the drug group (p=.083, η²=.033). Calcium decreased after surgery (p<.001, η²=.202), with a greater reduction in the drug group (interaction: p=.067, η²=.022). The AST/ALT ratio showed a non-significant increase post-operatively and was slightly higher in the drug group (p=.074, η²=.008 for both).

| **Supplementary Table S3. Perioperative blood samples were collected and analyzed for colorectal cancer patients from the COMPIT2 study** | | | | | | | | | |
| --- | --- | --- | --- | --- | --- | --- | --- | --- | --- |
| **Normal Range** | **Measure** | **Pre-op Mean ± SD** | | **Post-op Mean ± SD** | | **Surgery**  **(p-value, effect size)** | **Treatment**  **(p-value, FDR-adjusted p-value^a^, effect size)** | **Treatment**  **Effect**  **(Δ [95% CI])** | **Interaction**  **(p-value, FDR-adjusted p-value^a^, effect size)** |
|  |  | **Drugs**  (n=20) | **Placebo**  (n=17) | **Drugs**  (n=20) | **Placebo**  (n=17) |  |  |  |  |
| 1<NLR<2 | NLR | 3.01±1.74 | 4.11±2.68 | 10.12±7.13 | 14.61±10.04 | p<.001, η²=.326 | p=.083, NS, η²=.033 | −2.79 [−5.99, 0.39] | NS, NS, - |
| 50-70% | %Neutrophils | 64.90±9.94 | 65.93±10.76 | 81.53±8.93 | 82.85±9.18 | p<.001, η²=.438 | NS, NS, - | −1.17 [−6.1, 3.76] | NS, NS, - |
| 20-40% | %Lymphocytes | 23.91±9.45 | 22.22±9.85 | 11.75±6.79 | 9.53±7.00 | p<.001, η²=.363 | NS, NS, - | 1.96 [−2.39, 6.3] | NS, NS, - |
| 2-8% | %Monocytes | 7.55±2.66 | 8.28±2.97 | 5.78±2.64 | 6.32±2.49 | p<.001, η²=.111 | NS, NS, - | −0.63 [−2.12, 0.86] | NS, NS, - |
| 1-4% | %Eosinophils | 2.63±1.71 | 2.64±2.12 | 0.48±0.51 | 0.74±0.96 | p<.001, η²=.338 | NS, NS, - | -0.13 [−0.83, 0.59] | NS, NS, - |
| 0-1% | %Basophils | 0.57±0.25 | 0.61±0.29 | 0.19±0.13 | 0.19±0.14 | p<.001, η²=.470 | NS, NS, - | −0.01 [−0.14, 0.11] | NS, NS, - |
|  |  | **Drugs**  (n=19-20) | **Placebo**  (n=15-16) | **Drugs**  (n=19-20) | **Placebo**  (n=15-16) |  |  |  |  |
| 0.7-1.2 mg/dL | Creatinine mg/dl | 0.82±0.18 | 0.80±0.16 | 0.82±0.29 | 0.77±0.22 | NS, - | NS, NS, - | 0.03 [−0.1, 0.17] | NS, NS, - |
| 70-100 mg/dL | Glucose mg/dl | 108.55±34.82 | 104.00±27.60 | 146.15±35.51 | 128.12±36.68 | p<.001, η²=.172 | NS, NS, - | 11.29 [−8.22, 30.8] | NS, NS, - |
| 3.5-5.1 meq/L | Potassium meq/l | 4.13±0.35 | 4.06±0.53 | 4.27±0.38 | 4.20±0.44 | NS | NS, NS, - | 0.08 [−0.14, 0.3] | NS, NS, - |
| 135-145 meq/l | Sodium meq/l | 139.00±2.38 | 139.37±2.53 | 137.95±2.33 | 138.56±2.00 | p=.022, η²=.040 | NS, NS, - | −0.49 [−1.87, 0.88] | NS, NS, - |
| 8.6-10.3 mg/dL | Calcium mg/dl | 9.09±0.63 | 8.93±0.65 | 8.28±0.60 | 8.52±0.55 | p<.001, η²=.202 | NS, NS, - | −0.04 [−0.41, 0.33] | p=.067, NS, η²=.022 |
|  |  | **Drugs**  (n=16) | **Placebo**  (n=12-13) | **Drugs**  (n=16) | **Placebo**  (n=12-13) |  |  |  |  |
| 5-38 U/L | SGOT (AST) IU/l | 25.94±11.85 | 26.83±13.74 | 25.37±9.24 | 26.25±10.22 | NS, - | NS, NS, - | −0.88 [−8.66, 6.89] | NS, NS, - |
| 4-41 U/L | SGPT (ALT) IU/l | 21.94±21.46 | 28.15±30.86 | 18.50±14.79 | 23.46±28.87 | p=.01, η²=.007 | NS, NS, - | −5.59 [−23.88, 12.7] | NS, NS, - |
| <1 | AST/ALT | 1.69±1.49 | 1.17±0.58 | 1.69±1.30 | 1.58±1.00 | p=.074, η²=.008 | NS, NS, - | 0.31 [−0.59, 1.22] | p=.074, NS, η²=.008 |
| 3.4-4.8 g/dl | Albumin g/dl | 4.04±0.50 | 4.21±0.50 | 3.39±0.48 | 3.52±0.65 | p<.001, η²=.290 | NS, NS, - | −0.15 [−0.54, 0.24] | NS, NS, - |
|  |  | **Drugs**  (n=9) | **Placebo**  (n=8) | **Drugs**  (n=9) | **Placebo**  (n=8) |  |  |  |  |
| 2.0-3.5 g/dL | Globulin g/dl | 2.71±0.37 | 2.77±0.48 | 2.20±0.41 | 2.36±0.58 | p<.001, η²=.216 | NS, NS, - | −0.11 [−0.57, 0.34] | NS, NS, - |

SD = Standard Deviation. NS indicates p ≥ 0.1; Borderline p-values (.05 ≤ p < .10) are reported for transparency, but significance threshold is α = 0.05. ^a^Adjusted using the Benjamini–Hochberg false discovery rate (FDR) method. Effect sizes are reported as partial eta squared (η²) for repeated measures ANOVA. CI = Confidence Interval. Treatment Effect (Δ [95% CI]) represents the model-based estimate of the mean difference (Drugs − Placebo) with 95% CIs, reported regardless of statistical significance.

**Pancreatic cancer RCT- 1 month postoperative**

Blood samples from a subset of 22 PC patients (BCPC study) were analyzed for differences between the day before surgery and 1-month postoperatively. Among the blood markers examined, significant interaction effects between surgery and treatment were observed for creatinine, potassium, and calcium levels. For creatinine, a significant interaction emerged (p=.023, η²=.076), with levels slightly decreasing postoperatively in the drug-treated group, while increasing in the placebo group. For potassium and calcium, similar interactions were found (p=.027, η²=.084; p=.020, η²=.097). Despite these differences, all values remained within normal physiological ranges (Supplementary Table S4).

| **Supplementary Table S4. 1 Month post-op blood samples were collected and analyzed from 24 patients of BCPC study** | | | | | | | | | |
| --- | --- | --- | --- | --- | --- | --- | --- | --- | --- |
| **Normal Range** | **Criteria** | **Pre-op Mean ± SD** | | **Post-op (Day ~+30) Mean ± SD** | | **Surgery**  **(p-value, effect size)** | **Treatment**  **(p-value, FDR-adjusted p-value^a^, effect size)** | **Treatment**  **Effect**  **(Δ [95% CI])** | **Interaction**  **(p-value, FDR-adjusted p-value^a^, effect size)** |
|  |  | **Drugs**  (n=10-11) | **Placebo**  (n=9-11) | **Drugs**  (n=10-11) | **Placebo**  (n=9-11) |  |  |  |  |
| 1<NLR<2 | NLR | 3.25±1.73 | 2.40±1.72 | 6.66±7.75 | 6.10±9.59 | p=.09, η²=.08 | NS, NS, - | 0.7 [−3.3, 4.7] | NS, NS, - |
| 50-70% | Neutrophil % | 66.67±12.83 | 56.68±12.57 | 65.84±17.28 | 65.47±16.64 | NS, - | NS, NS, - | 5.18 [−4.15, 14.51] | NS, NS, - |
| 20-40% | Lymphocytes % | 23.05±13.24 | 32.21±12.08 | 21.37±13.98 | 23.84±12.54 | NS, - | NS, NS, - | −5.81 [−13.89, 2.26] | NS, NS, - |
| 2-8% | Monocytes % | 7.53±2.22 | 8.84±4.00 | 9.38±2.83 | 8.02±4.03 | NS, - | NS, NS, - | 0.02 [−2.31, 2.36] | NS, NS, - |
| 1-4% | Eosinophil % | 2.24±2.03 | 1.54±0.98 | 2.52±2.03 | 2.06±2.19 | NS, - | NS, NS, - | 0.57 [−0.7, 1.85] | NS, NS, - |
| 0-1% | Basophil % | 0.49±0.36 | 0.74±0.36 | 0.68±0.38 | 0.72±0.64 | NS, - | NS, NS, - | −0.14 [−0.42, 0.14] | NS, NS, - |
| 0.7-1.2 mg/dL | Creatinine mg/dl | 0.82±0.26 | 0.72±0.27 | 0.67±0.26 | 0.84±0.22 | NS, - | NS, NS, - | −0.03 [−0.22, 0.16] | p=.023, NS, η²=.076 |
| 70-100 mg/dL | Glucose mg/dl | 118.09±25.51 | 131.64±33.66 | 128.91±48.19 | 121.00±22.37 | NS, - | NS, NS, - | −2.82 [−23.96, 18.32] | NS, NS, - |
| 3.5-5.1 meq/L | Potassium meq/l | 4.59±0.48 | 4.31±0.75 | 4.14±0.39 | 4.53±0.63 | NS, - | NS, NS, - | −0.05 [−0.48, 0.37] | p=.027, NS, η²=.084 |
| 135-145 meq/l | Sodium meq/l | 139.09±1.22 | 139.73±3.35 | 137.91±5.80 | 139.73±3.00 | NS, - | NS, NS, - | −1.23 [−3.66, 1.2] | NS, NS, - |
| 8.6-10.3 mg/dL | Calcium mg/dl | 9.55±0.63 | 9.39±0.26 | 8.76±0.86 | 9.52±0.85 | p=.082, η²=.051 | NS, NS, - | −0.29 [−0.78, 0.19] | p=.020, NS, η²=.097 |
| 5-38 U/L | SGOT (AST) IU/l | 33.70±20.25 | 71.45±78.13 | 34.80±16.39 | 40.36±34.61 | NS, - | NS, NS, - | −21.66 [−53.01, 9.7] | NS, NS, - |
| 4-41 U/L | SGPT (ALT) IU/l | 42.80±27.84 | 76.36±124.10 | 36.90±33.55 | 45.00±58.42 | NS, - | NS, NS, - | −20.83 [−71.59, 29.93] | NS, NS, - |
| 3.4-4.8 g/dl | Albumin g/dl | 3.64±0.66 | 3.62±0.57 | 3.24±0.82 | 3.61±0.72 | NS, - | NS, NS, - | −0.18 [−0.7, 0.33] | NS, NS, - |
| 2.0-3.5 g/dL | Globulin g/dl | 2.73±0.57 | 2.81±0.58 | 2.99±0.63 | 2.79±0.86 | NS, - | NS, NS, - | 0.06 [−0.45, 0.57] | NS, NS, - |
| <1 | AST/ALT | 1.00±0.47 | 1.36±0.50 | 1.26±0.46 | 1.25±0.53 | NS, - | NS, NS, - | −1.18 [−0.56, 0.21] | NS, NS, - |

SD = Standard Deviation. NS indicates p ≥ 0.1; Borderline p-values (.05 ≤ p < .10) are reported for transparency, but significance threshold is α = 0.05. ^a^Adjusted using the Benjamini–Hochberg false discovery rate (FDR) method. Effect sizes are reported as partial eta squared (η²) for repeated measures ANOVA. CI = Confidence Interval. Treatment Effect (Δ [95% CI]) represents the model-based estimate of the mean difference (Drugs − Placebo) with 95% CIs, reported regardless of statistical significance. One sample was excluded from analysis of SGOT (AST) and SGPT (ALT) due to outlier values (>3 SD from the mean).

**Long-term outcomes of eight-year follow-up:**

**Colorectal cancer RCT (COMPIT1)**

| Figure S1. Kaplan-Meier curve for 8-year DFS in the protocol-compliant population  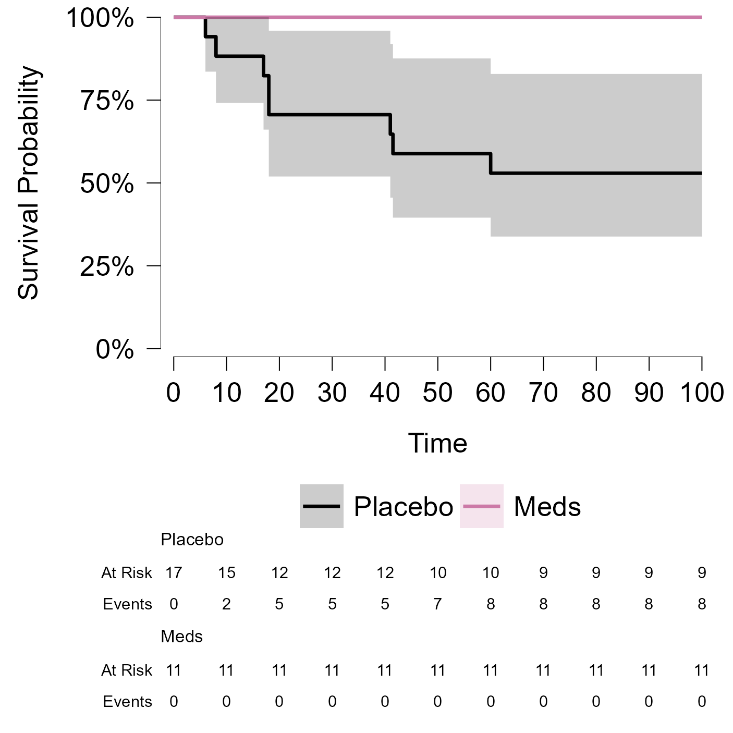  Time in months. Kaplan–Meier estimates of DFS in protocol-compliant CRC patients. No recurrences in the protocol-compliant drug-treated colorectal cancer patients; 0/11vs. 8/17 in placebo (p=.01). HR could not be estimated due to zero events in the treatment | Figure S2. Kaplan-Meier curve for 8-years OS in the protocol-compliant population  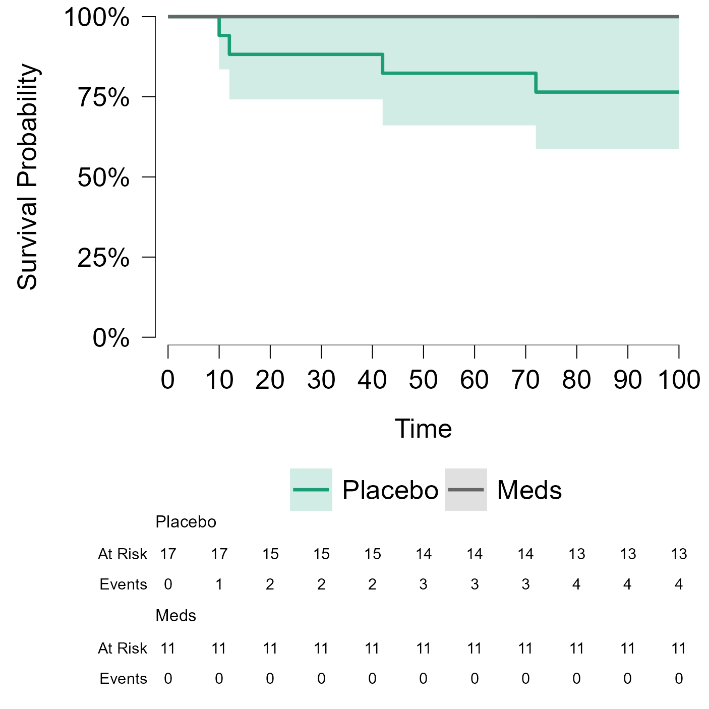    Time in months. No deaths in the protocol-compliant drug-treated group; 0/11 vs. 4/17 in placebo (p=.091); not statistically significant. HR could not be estimated due to zero events in the treatment group. |
| --- | --- |

Supplementary Table S5. Cox proportional hazards model for DFS, ITT patients, including treatment and covariates

| *Cox Proportional Hazards Summary Table* | | | | | | | | | | |  | |  | |  | |  | |  | |  | |
| --- | --- | --- | --- | --- | --- | --- | --- | --- | --- | --- | --- | --- | --- | --- | --- | --- | --- | --- | --- | --- | --- | --- |
| Model | | Log Lik. | | df | | AIC | | BIC | | |  | |  | |  | |  | |  | |  | |
| H₀ |  | -36.178 |  | 0 |  | 72.356 |  | 72.356 |  |  | |  | |  | |  | |  | |  | |  |
| H₁ |  | -32.874 |  | 4 |  | 73.748 |  | 75.339 |  |  | |  | |  | |  | |  | |  | |  |
|  | | | | | | | | | | |  | |  | |  | |  | |  | |  | |
| *Note.*  1 observations ommited due to missing values. | | | | | | | | | | |  | |  | |  | |  | |  | |  | |
| *Note.*  32 observations with 11 events. | | | | | | | | | | |  | |  | |  | |  | |  | |  | |
| *Cox Proportional Hazards Estimates Table* | | | | | | | | | | | | | | | | | | | | | | |
|  | | | | | | | | 95% CI | | | | | | |  | | | | | | | |
| Model | |  | | Estimate | | Standard Error | | Lower | | | Upper | | | | z | | | | p | | | |
| H₁ |  | Treatment (Meds) |  | -1.864 |  | 0.843 |  | -3.517 |  | -0.211 | |  | | -2.21 | |  | | 0.027 | |  | |  |
|  |  | Age |  | -0.018 |  | 0.029 |  | -0.076 |  | 0.04 | |  | | -0.608 | |  | | 0.543 | |  | |  |
|  |  | BMI |  | -0.053 |  | 0.079 |  | -0.207 |  | 0.101 | |  | | -0.672 | |  | | 0.501 | |  | |  |
|  |  | Tstage |  | -0.233 |  | 0.326 |  | -0.873 |  | 0.406 | |  | | -0.715 | |  | | 0.474 | |  | |  |
|  | | | | | | | | | | | | | | | | | | | | | | |
| *Hazard Ratios Estimates Table* | | | | | | | | | | |  | |  | |  | |  | |  | |  | |
|  | | | | | | 95% CI | | | | |  | |  | |  | |  | |  | |  | |
| Model | |  | | Hazard Ratio | | Lower | | Upper | | |  | |  | |  | |  | |  | |  | |
| H₁ |  | Treatment (Meds) |  | 0.155 |  | 0.03 |  | 0.81 |  |  | |  | |  | |  | |  | |  | |  |
|  |  | Age |  | 0.982 |  | 0.927 |  | 1.041 |  |  | |  | |  | |  | |  | |  | |  |
|  |  | BMI |  | 0.948 |  | 0.813 |  | 1.107 |  |  | |  | |  | |  | |  | |  | |  |
|  |  | Tstage |  | 0.792 |  | 0.418 |  | 1.501 |  |  | |  | |  | |  | |  | |  | |  |
|  | | | | | | | | | | |  | |  | |  | |  | |  | |  | |

Supplementary Table S6. Cox proportional hazards model for DFS, protocol-compliant patients, including treatment and covariates

| *Cox Proportional Hazards Summary Table* | | | | | | | | | | |  | |  | |  | |  | |  | |  | |
| --- | --- | --- | --- | --- | --- | --- | --- | --- | --- | --- | --- | --- | --- | --- | --- | --- | --- | --- | --- | --- | --- | --- |
| Model | | Log Lik. | | df | | AIC | | BIC | | |  | |  | |  | |  | |  | |  | |
| H₀ |  | -25.218 |  | 0 |  | 50.435 |  | 50.435 |  |  | |  | |  | |  | |  | |  | |  |
| H₁ |  | -19.559 |  | 4 |  | 47.118 |  | 47.436 |  |  | |  | |  | |  | |  | |  | |  |
|  | | | | | | | | | | |  | |  | |  | |  | |  | |  | |
| *Note.*  1 observations ommited due to missing values. | | | | | | | | | | |  | |  | |  | |  | |  | |  | |
| *Note.*  27 observations with 8 events. | | | | | | | | | | |  | |  | |  | |  | |  | |  | |
| *Cox Proportional Hazards Estimates Table* | | | | | | | | | | | | | | | | | | | | | | |
|  | | | | | | | | 95% CI | | | | | | |  | | | | | | | |
| Model | |  | | Estimate | | Standard Error | | Lower | | | Upper | | | | z | | | | p | | | |
| H₁ |  | Treatment (Meds) |  | -21.381 |  | 14139.206 |  | -27733.7 |  | 27690.953 | |  | | -0.002 | |  | | 0.999 | |  | |  |
|  |  | Age |  | -0.032 |  | 0.043 |  | -0.115 |  | 0.052 | |  | | -0.741 | |  | | 0.459 | |  | |  |
|  |  | BMI |  | -0.037 |  | 0.093 |  | -0.219 |  | 0.145 | |  | | -0.4 | |  | | 0.689 | |  | |  |
|  |  | Tstage |  | -0.2 |  | 0.432 |  | -1.047 |  | 0.647 | |  | | -0.462 | |  | | 0.644 | |  | |  |
|  | | | | | | | | | | | | | | | | | | | | | | |
| *Hazard Ratios Estimates Table* | | | | | | | | | | |  | |  | |  | |  | |  | |  | |
|  | | | | | | 95% CI | | | | |  | |  | |  | |  | |  | |  | |
| Model | |  | | Hazard Ratio | | Lower | | Upper | | |  | |  | |  | |  | |  | |  | |
| H₁ |  | Treatment (Meds) |  | 5.178×10^-10^ |  | 0 |  | ∞ |  |  | |  | |  | |  | |  | |  | |  |
|  |  | Age |  | 0.969 |  | 0.891 |  | 1.053 |  |  | |  | |  | |  | |  | |  | |  |
|  |  | BMI |  | 0.964 |  | 0.803 |  | 1.156 |  |  | |  | |  | |  | |  | |  | |  |
|  |  | Tstage |  | 0.819 |  | 0.351 |  | 1.911 |  |  | |  | |  | |  | |  | |  | |  |
|  | | | | | | | | | | |  | |  | |  | |  | |  | |  | |

Supplementary Table S7. Cox proportional hazards model for OS, ITT patients, including treatment and covariates

|  | | | | | |  |  |  |  |  |  |  |  |  |  |
| --- | --- | --- | --- | --- | --- | --- | --- | --- | --- | --- | --- | --- | --- | --- | --- |
| *Cox Proportional Hazards Summary Table* | | | | | | | | | |  |  |  |  |  |  |
| Model | | Log Lik. | | df | | AIC | | BIC | |  |  |  |  |  |  |
| H₀ |  | -19.874 |  | 0 |  | 39.747 |  | 39.747 |  |  |  |  |  |  |  |
| H₁ |  | -19.512 |  | 4 |  | 47.024 |  | 46.191 |  |  |  |  |  |  |  |
|  | | | | | | | | | |  |  |  |  |  |  |
| *Note.*  3 observations ommited due to missing values. | | | | | | | | | |  |  |  |  |  |  |
| *Note.*  30 observations with 6 events. | | | | | | | | | |  |  |  |  |  |  |
| *Cox Proportional Hazards Estimates Table* | | | | | | | | | | | | | | | |
|  | | | | | | | | 95% CI | | | |  | | | |
| Model | |  | | Estimate | | Standard Error | | Lower | | Upper | | z | | p | |
| H₁ |  | Treatment (Meds) |  | -0.439 |  | 0.928 |  | -2.257 |  | 1.38 |  | -0.473 |  | 0.636 |  |
|  |  | BMI |  | 0.027 |  | 0.087 |  | -0.144 |  | 0.197 |  | 0.307 |  | 0.759 |  |
|  |  | Age |  | 0.005 |  | 0.039 |  | -0.071 |  | 0.081 |  | 0.127 |  | 0.899 |  |
|  |  | Tstage |  | 0.176 |  | 0.491 |  | -0.786 |  | 1.138 |  | 0.358 |  | 0.72 |  |
|  | | | | | | | | | | | | | | | |
| *Hazard Ratios Estimates Table* | | | | | | | | | |  |  |  |  |  |  |
|  | | | | | | 95% CI | | | |  |  |  |  |  |  |
| Model | |  | | Hazard Ratio | | Lower | | Upper | |  |  |  |  |  |  |
| H₁ |  | Treatment (Meds) |  | 0.645 |  | 0.105 |  | 3.974 |  |  |  |  |  |  |  |
|  |  | BMI |  | 1.027 |  | 0.866 |  | 1.218 |  |  |  |  |  |  |  |
|  |  | Age |  | 1.005 |  | 0.931 |  | 1.085 |  |  |  |  |  |  |  |
|  |  | Tstage |  | 1.192 |  | 0.456 |  | 3.12 |  |  |  |  |  |  |  |
|  | | | | | | | | | |  |  |  |  |  |  |

Supplementary Table S8. Cox proportional hazards model for OS, protocol-compliant patients, including treatment and covariates

|  | | | | | |  |  |  |  |  |  |  |  |  |  |
| --- | --- | --- | --- | --- | --- | --- | --- | --- | --- | --- | --- | --- | --- | --- | --- |
| *Cox Proportional Hazards Summary Table* | | | | | | | | | |  |  |  |  |  |  |
| Model | | Log Lik. | | df | | AIC | | BIC | |  |  |  |  |  |  |
| H₀ |  | -12.951 |  | 0 |  | 25.902 |  | 25.902 |  |  |  |  |  |  |  |
| H₁ |  | -9.264 |  | 4 |  | 26.529 |  | 24.074 |  |  |  |  |  |  |  |
|  | | | | | | | | | |  |  |  |  |  |  |
| *Note.*  1 observations ommited due to missing values. | | | | | | | | | |  |  |  |  |  |  |
| *Note.*  27 observations with 4 events. | | | | | | | | | |  |  |  |  |  |  |
| *Cox Proportional Hazards Estimates Table* | | | | | | | | | | | | | | | |
|  | | | | | | | | 95% CI | | | |  | | | |
| Model | |  | | Estimate | | Standard Error | | Lower | | Upper | | z | | p | |
| H₁ |  | Treatment (Meds) |  | -20.387 |  | 18342.771 |  | -35971.6 |  | 35930.782 |  | -0.001 |  | 0.999 |  |
|  |  | BMI |  | 0.023 |  | 0.1 |  | -0.174 |  | 0.22 |  | 0.225 |  | 0.822 |  |
|  |  | Age |  | -0.026 |  | 0.05 |  | -0.124 |  | 0.072 |  | -0.52 |  | 0.603 |  |
|  |  | Tstage |  | 18.756 |  | 11382.033 |  | -22289.6 |  | 22327.13 |  | 0.002 |  | 0.999 |  |
|  | | | | | | | | | | | | | | | |
| *Hazard Ratios Estimates Table* | | | | | | | | | |  |  |  |  |  |  |
|  | | | | | | 95% CI | | | |  |  |  |  |  |  |
| Model | |  | | Hazard Ratio | | Lower | | Upper | |  |  |  |  |  |  |
| H₁ |  | Treatment (Meds) |  | 1.400×10^-9^ |  | 0 |  | ∞ |  |  |  |  |  |  |  |
|  |  | BMI |  | 1.023 |  | 0.84 |  | 1.246 |  |  |  |  |  |  |  |
|  |  | Age |  | 0.974 |  | 0.883 |  | 1.075 |  |  |  |  |  |  |  |
|  |  | Tstage |  | 1.398×10^+8^ |  | 0 |  | ∞ |  |  |  |  |  |  |  |
|  | | | | | | | | | |  |  |  |  |  |  |


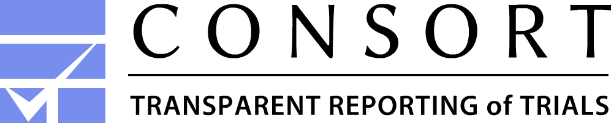
Supplementary Figure S3. CONSORT Diagram Across the Four Randomized Trials (Completed and Ongoing)

**CONSORT 2010 Flow Diagram**

## Enrollment

Assessed for eligibility (BC: n=99; COMPIT1: n=129; COMPIT2: n=92, PC: n=93)

Excluded (BC: n=61; COMPIT1: n=95; COMPIT2: n=46, PC: n=63)

♦  Not meeting inclusion criteria (n=225)

♦  Declined to participate (n=40)

♦  Other reasons (n=0)

Randomized (BC: n=38; COMPIT1: n=34; COMPIT2: n=46; PC: n=30)

Lost to DFS and OS follow-up in COMPIT1 trial (n=2)

Lost to blood measures follow-up in COMPIT1, COMPIT2, and PC (missing samples) (n=8)

Lost to DFS and OS follow-up in COMPIT1 trial (n=2)

Lost to blood measures follow-up in COMPIT1, COMPIT2, and PC (missing samples) (n=6)

Allocated to drug intervention (n=76)

♦ Received allocated intervention (n=76)

♦ Did not receive allocated intervention (n=0)

Allocated to placebo (n=72)

♦ Received allocated intervention (n=72)

♦ Did not receive allocated intervention (n=0)

## Allocation

## Follow-Up

## Analysis

♦ AEs analysis (ITT: n=76; Protocol-compliant: n=58)

♦ Blood samples analysis (n=49)

♦ DFS analysis (ITT: n=15; Protocol-compliant: n=11)

♦ OS analysis (ITT: n=14; Protocol-compliant: n=11)

♦ AEs analysis (ITT: n=72; Protocol-compliant: n=63)

♦ Blood samples analysis (n=47)

♦ DFS analysis (ITT: n=18; Protocol-compliant: n=17)

♦ OS analysis (ITT: n=17; Protocol-compliant: n=17)

Breast cancer (BC) and COMPIT1 colorectal cancer trials are completed and have published CONSORT diagrams; COMPIT2 colorectal cancer and pancreatic cancer (PC) trials are ongoing. Only COMPIT1 included a follow-up phase. Final CONSORT diagrams and exact screening logs will be provided upon trial completion.

Supplementary Statistical Analysis Plan (SAP)

**Endpoints**

- **Primary:** Blood measures (surgery morning and the day after surgery), and perioperative adverse events until 30 days postoperative.
- **Secondary/exploratory:** DFS/OS.

**Populations**

- Breast cancer: n=38; COMPIT1 colorectal cancer: n=34; COMPIT2 colorectal cancer: n=46; Pancreatic cancer: n=30)
- ITT: all randomized.
- Non Protocol-compliant: consuming less than 60% of pills throughout the treatment period, or consuming less than 75% (3 out of 4) of pills in either of the following periods: (i) on the day before surgery and the day of surgery, or (ii) during the first two days following surgery.

**Analyses**

- Blood measures: 2x2 RM-ANOVA and linear mixed model (LMM) (treatment, time (surgery), interaction; subject random intercept).
- AEs: counts, %, Fisher’s exact/chi-square; report RD, RR, 95% CI.
- DFS/OS: Kaplan–Meier, log-rank, adjusted and unadjusted Cox.

**Multiplicity**

- Benjamini–Hochberg FDR for blood measures.

**Missing data**

- LMM for blood measures.

**Software**

- JASP 0.18.3.0 software.
